# Rational design of novel benzisoxazole derivatives with acetylcholinesterase inhibitory and serotoninergic 5-HT_4_ receptors activities for the treatment of Alzheimer’s disease

**DOI:** 10.1038/s41598-020-59805-7

**Published:** 2020-02-20

**Authors:** Julien Lalut, Hugo Payan, Audrey Davis, Cédric Lecoutey, Rémi Legay, Jana Sopkova-de Oliveira Santos, Sylvie Claeysen, Patrick Dallemagne, Christophe Rochais

**Affiliations:** 1 Normandie Univ, UNICAEN, Centre d’Etudes et de Recherche sur le Médicament de Normandie (CERMN), Caen, France; 2 IGF, Univ. Montpellier, CNRS, INSERM, Montpellier, France

**Keywords:** Drug discovery and development, Structure-based drug design, Medicinal chemistry, Alzheimer's disease

## Abstract

A rigidification strategy was applied to the preclinical candidate donecopride, an acetylcholinesterase inhibitor possessing 5-HT_4_R agonist activity. Inspired by promising bioactive benzisoxazole compounds, we have conducted a pharmacomodulation study to generate a novel series of multitarget directed ligands. The chemical synthesis of the ligand was optimized and compounds were evaluated *in vitro* against each target and in cellulo. Structure-activity relationship was supported by docking analysis in human acetylcholinesterase binding site. Among the synthesized compounds, we have identified a novel hybrid **32a** (3-[2-[1-(cyclohexylmethyl)-4-piperidyl]ethyl]-4-methoxy-1,2-benzoxazole) able to display nanomolar acetylcholinesterase inhibitory effects and nanomolar K*i* for 5-HT_4_R.

## Introduction

As people live longer, the number of people with dementia in low- and middle-income countries and estimates of prevalence raised rapidly and are constantly being revised upwards. In 2018, 50 million people are living with dementia worldwide and this number will increase to 152 million by 2050^1^. Currently ranked in the first places leading causes of death for older people, the most common dementia, Alzheimer’s disease (AD), is a progressive and irreversible brain disorder characterized by a slowly decline in memory and thinking skills up to the inability to carry out the simplest everyday tasks^2^. The histological diagnosis of AD is based on the presence in abnormally high quantities of two types of lesions in the central nervous system: the senile plaques formed by aggregation of β-amyloid protein (Aβ) and the neurofibrillary tangles due to hyperphosphorylation of tau protein^3^.

For more than 20 years, therapeutic approaches, focused on the neuronal dysfunction, only allowed to develop treatment with symptomatic benefits for patients, by regulating neurotransmitters^4^. Except memantine, an NMDA receptor antagonist approved to regulate the glutamatergic activity, the three other drugs authorized by the FDA (donepezil, rivastigmine and galanthamine) are acetylcholinesterase (AChE) inhibitors. Usually used to treat mild to moderate forms of AD, AChE inhibitors (AChEIs) reduce, by inhibiting the catalytic active site (CAS) of the enzyme, the excessive degradation of acetylcholine (ACh), a neurotransmitter involved in many physiological processes including memory and learning and thus restore cholinergic transmission. However, these drugs only work for a limited time and do not change the underlying disease process.

The multifactorial origin of AD has led researchers to develop a new approach that could exert a disease-modifying effect, aimed at slowing down, stopping or reversing the progression of AD, by direct actions on pathophysiological pathways as early as possible^5^. While disease modifying strategies have not yet allowed any marketing authorization despite many preclinical and clinical trials (ineffectiveness, side effects, lack of selectivity)^6^ the development of a selective compound against a single AD target does not seem to be the best-suited strategy to the complex AD puzzle.

In order to target several molecular causes implicated in the pathogenesis of AD disease, a new concept emerged about 10 years ago: the development of Multi-Target Directed Ligands (MTDLs)^7,8^. This innovative and promising drug discovery approach allows the design of ligands targeting, from a single active compound, several pharmacological targets involved in the pathogenesis of a same disease and showing a more synergistic effect. Compared to traditional pharmacological approaches (multiple-medication therapy or multiple-compound medication) used for multifactorial diseases, this concept has advantages such as a simplified clinical development (pharmacokinetic study and optimization of ADME-Tox properties) and a lower risk of drug-drug interactions or poor patient compliance avoided. Based on the benefit that could be the synergy of several therapeutic effects obtained from different pharmacological targets on clinical efficacy, Morphy *et al*. have nevertheless reported the difficulty of adjusting the ratio of activities towards the various targets^7^.

Many MTDLs have been developed for the inhibition of AChE, and more particularly dual-binding site (DBS) AChE inhibitors targeting simultaneously the two sites of the enzyme, the CAS and the peripheral anionic site (PAS)^9,10^. Indeed, it has been shown that the PAS could form a toxic complex with Aβ favoring the formation of fibrils and consequently their aggregation into senile plaques^11–13^.

The interaction with both sites allows the restoration of the cholinergic deficit by blocking the CAS activity and, in addition to preventing the access of ACh to the active site, the inhibition of PAS disrupts amyloid aggregation. Other MTDLs have been developed by adding another activity to the AChE inhibition (BACE1 or MAOs inhibitors, metal chelators, antioxidants…)^14,15^ or by combining different rational therapeutic activities without targeting the enzyme^16^.

Our laboratory has published data on several chemical families of MTDLs useful for treating AD^17–19^, Among them, donecopride, based on a structural compromise between donepezil, an AChEI drug, and RS67333, a serotonin subtype 4 receptor (5-HT_4_R) agonist, seems to be a promising drug candidate with excellent *in vitro* activities (*h*AChE IC_50_ = 16 nM and 5-HT_4_R *K*_i_ = 8.5 nM, 48% of control agonist response) and *in vivo* effects (improvement of memory performances on the object recognition test in mice)^20^. Donecopride, the first MTDL described in the literature associating these both complementary activities, is actually engaged in preclinical trials.

Located in the CNS, 5-HT_4_Rs are involved in cognitive functions (learning, memory…) and their stimulation leads to an improvement in the release of neurotransmitters and an increase in their concentration in brain areas^21–23^. The partial agonist RS67333 showed an improvement of cognitive performances in animals^24^. The increased release of ACh in combination with decreased breakdown due to inhibition of AchE is particularly interesting to counteract memory deficits as in AD. Additionally, activation of 5-HT_4_Rs promotes the non-amyloidogenic cleavage of amyloid precursor protein (APP), producing the soluble and neurotrophic fragment sAPPα to the detriment of Aβ production^25^. We have earlier demonstrated that the co-modulation of those two targets (AChE inhibition and 5-HT_4_R agonism with two reference compounds) appears synergistic in *in vivo* models of memory deficit^26^. The published results obtained with donecopride demonstrated the potential of this strategy. We would also like to explore novel heterocyclic scaffold in order to verify if this combination of activities is transposable to novel chemical series that could combine a symptomatic action (restoration of the cholinergic activity) and a disease-modifying effect (promotion of sAPPα, inhibition of the AChE-induced Aβ aggregation) that seems to be a relevant approach to treat AD.

**Figure 1:**
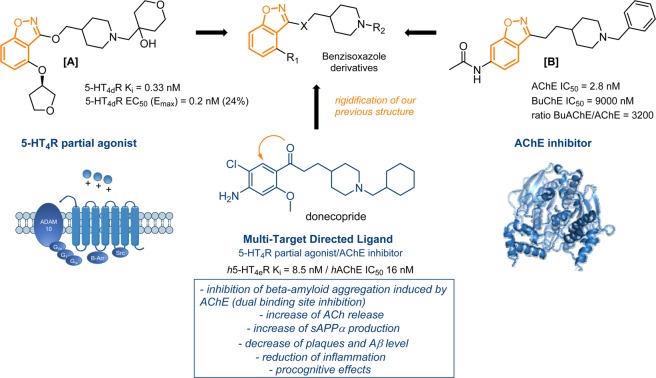
Rational design of novel MTDLs with acetylcholinesterase inhibition and serotoninergic 5-HT_4_ receptors activity, guided by previous works in the literature.

Villalobos *et al*. have reported the development of a series of *N*-benzylpiperidine benzisoxazoles as potent and selective AChEI^27^. While keeping a benzylated piperidine, the benzisoxazole ring was investigated as a suitable bioisostere to replace the benzoylamino scaffold present in a class of AChE inhibitors previously described^28^. All benzisoxazoles synthesized displayed submicromolar *h*AChE IC50s. The most promising *N*-acetyl derivative **[B]** (Fig. 1) showed an excellent AChE inhibition activity with an IC_50_ of 2.8 nM and a favorable profile *in vivo* (a dose-dependent elevation of ACh in mouse and a reversion of amnesia in a mouse passive avoidance model). According to the docking realized for this AChEI, the compound seems able to interact with the PAS of the enzyme. More recently, Brodney *et al*. have identified 5-HT_4_R partial agonists with a similar benzisoxazole scaffold^29^. By exploring various alkoxyl substituents in position C3 of the benzisoxazole ring and two different substituents on the piperidine ring, the resulting compounds displayed a nanomolar affinity towards 5-HT_4_R. Many ADME parameters (clearance from human liver microsomes, passive permeability, cyclic AMP production, neuropharmacokinetic parameters…) have been studied and two compounds, among them the benzisoxazole **[A]** (Fig. 1) with a 5-HT_4_R *K*_i_ of 9.6 nM, were chosen to continue in preclinical trials.

On the basis of these encouraging results for the development of AChE inhibitors on one hand and 5-HT_4_R agonists on the other, we decided to synthesize new benzisoxazole MTDLs while keeping a structure close to donecopride (Fig. 1). Based on a structural compromise between the two chemical series already described, this work should allow us to rigidify the structure of the latter to study the impact of this new heterocycle on the two pharmacological targets.

## Results

### Chemistry

Many chemical reactions have been reported in literature concerning the formation of the 1,2-benzoxazole ring, substituted or not in position C3. To summarize some of them, we can quote non-exhaustively the concomitant formation of a C–C bond and an O–C bond by a [3 + 2] cycloaddition of nitrile oxides and arynes^30^, the formation of a O–N bond from readily accessible *ortho*-hydroxyaryl N–H ketimines through a N–Cl imine^31^ or the formation of a N=C bond *via* an intramolecular transoximation^32^.

**Figure 2:**
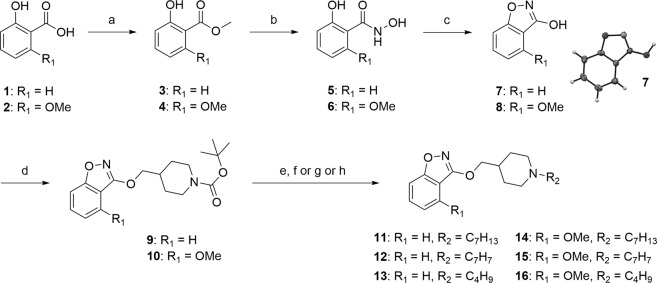
Synthesis of C3 substituted-benzisoxazole by a C–O bond with a hydrogen or a methoxy group in C4. Reagents and conditions: (**a**) conc. H_2_SO_4_, MeOH, reflux, 48 h, 92–94% (**b**) NH_2_OH.HCl, NaOH, H_2_O/dioxane (3:1), 1 h, rt then 15 h, 40 °C, 65–93% (**c**) CDI, dry THF, reflux, 3 h, 74–94% (**d**) *tert*-butyl 4-(hydroxymethyl)piperidine-1-carboxylate, DEAD, PPh_3_, dry THF, reflux, 19 h, 44–65% (**e**) TFA, CH_2_Cl_2_, rt, 1 h (**f**) bromomethylcyclohexane, K_2_CO_3_, DMF, 110 °C, 3 h, 22–45% (2 steps) (**g**) bromomethylbenzene, Et_3_N, CH_2_Cl_2_, rt, 15 h, 26–86% (2 steps) (**h**) 1-iodo-2-methyl-propane, Et_3_N, EtOH, reflux, 48 h, 23–36% (2 steps).

We first focused on the synthesis of compounds **11**–**16**, substituted by a C–O bond in position C3 and bearing a hydrogen or a methoxy group in position C4, from an intermediate *N*,2-dihydroxybenzamide. The latter can be obtained in particular according from the Angeli-Rimini reaction between an aldehyde and *N*-hydroxybenzenesulfonamide. In our case, the synthesis of hydroxamic acids **5** and **6** began by an esterification reaction of benzoic acids **1**–**2** under classical acidic conditions, followed by treatment of esters **3**–**4** with hydroxylamine in the presence of sodium hydroxide^33^. Cyclization of **5**–**6** with 1,1′-carbonyldiimidazole in anhydrous THF under reflux for 3 h led to build the benzisoxazole ring. The precipitation of the crude in water by addition of HCl allowed to afford derivatives **7**–**8** after suction filtration. The compound **7** was obtained in 94% yield and its structure was confirmed by X-ray diffraction of obtained crystals (Fig. 2). In the second case, the compound **8** was obtained in mixture with the compound **8′** having a 1,3-benzoxazole ring (see Supporting Information). This reaction was repeated several times and the expected product **8** with a 1,2-benzoxazole ring has nevertheless still been obtained as the major compound. The formation of the byproduct **8′** seems to be explained by the Lossen rearrangement, already described in the literature and allowing the conversion of an activated hydroxamic acid to isocyanate under the action of a base or a thermal activation^34^. The isocyanate can then easily react with an alcohol-containing compound. A reaction, according to the classical conditions described by Mitsunobu *et al*., then allows the conversion of the primary alcohols of the cyclized products **7**–**8** into ether links^35^. Compound **7** and the mixture **8**–**8′** (known ratio) were reacted with a hydroxylated piperidine chain in the presence of PPh_3_ and DEAD in dry THF to led to the derivative **9** in 44% yield and to the mixture **10**–**10′** in 79% yield. The separation of the both 1,2- and 1,3-benzoxazole **10**–**10′** was then carried out after this step (see Supporting Information). The piperidines **9**–**10** were deprotected under acidic conditions and we introduced several chains by an *N*-alkylation reaction using halogenated derivatives and a base according various conditions, leading to the desired compounds **11**–**16** in 22–45% yields over two steps (Fig. 2).

**Figure 3:**
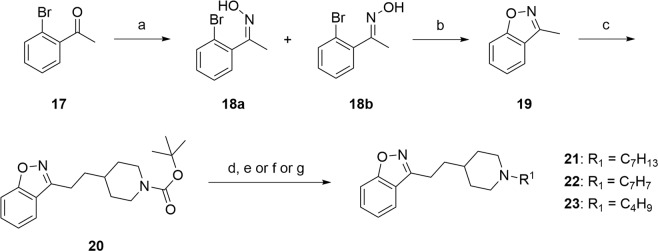
Synthesis of C3 substituted-benzisoxazole by a C-C bond. Reagents and conditions: (**a**) NH_2_OH.HCl, NaOAc, H_2_O/EtOH (3:2), 1 h, rt then 2 h, reflux, 90% (mixture of isomers *E*/*Z* 2:1) (**b**) *t*-BuONa, cat. DMEDA, cat. CuI, dry THF, 1 h, rt, 27% (**c**) LDA 1.0 M sol., dry THF, −78 °C then *tert*-butyl 4-(iodomethyl)piperidine-1-carboxylate, 1 h, −78 °C, 22% (**d**) TFA, CH_2_Cl_2_, rt, 1 h (**e**) bromomethylcyclohexane, K_2_CO_3_, DMF, 110 °C, 3 h, 50% (2 steps) (**f**) bromomethylbenzene, Et_3_N, CH_2_Cl_2_, rt, 15 h, 38% (2 steps) (**g**) 1-iodo-2-methyl-propane, Et_3_N, DMF, 110 °C, 3 h, 15% (2 steps).

We then decided to evaluate the influence of a methylene linker in position C3 instead of a C-O bond following a similar chemical strategy as that developed by Villalobos *et al*., namely the formation of the C-C bond by deprotonation of the 3-methyl-1,2-benzoxazole derivative using a lithiated base and subsequent reaction with an electrophile^27^. Synthesis of the expected methylated compound **19** began by a reaction between the *o*-bromoacetophenone and hydroxylamine in the presence of sodium acetate to obtain the mixture of oximes **18a**–**18b** (*E*/*Z*, 2:1) in 90% yield. A cyclization of these 2-bromophenyl ketoxime, engaged without separation of the both isomers according to the conditions reported by Udd and co-workers, allowed to form quickly and at room temperature the 3-methyl-1,2-benzoxazole **19**^36^. The overall yield was 27%, but the copper-catalyzed cyclization of *o*-brominated aromatic oximes was reported as being effective only for oximes of (*Z*) configuration (65% yield for the only *Z* isomer). Deprotonation of methylated compound **19** occurred at −78 °C in anhydrous THF using a lithium diisopropylamide solution and was followed by the addition of a (iodomethyl)piperidine chain to obtain the derivative **20**. Finally, the cleavage of the *tert*-butoxycarbonyl group was performed using trifluoroacetic acid and the deprotected piperidines was subsequently reacted with halogenated derivatives to afford the target compounds **21**–**23** in 15–50% yields over two steps (Fig. 3).

**Figure 4:**
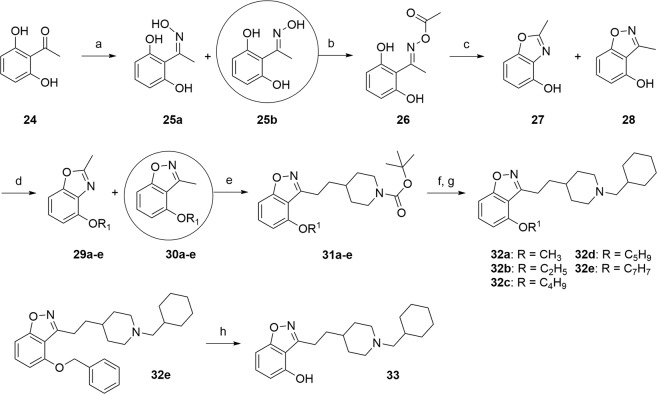
Synthesis of C3 substituted-benzisoxazole by a C-C bond with various ether groups or a hydroxyl group in C4. Reagents and conditions: (**a**) NH_2_OH.HCl, NaOAc, H_2_O/EtOH (3:2), 1 h, rt then 3 h, reflux, quantitative yield (mixture of isomers *E*/*Z* 4:1) (**b**) Ac_2_O, 1 h, rt, 83% (**c**) pyridine, 1 h, reflux, 71% (mixture **27**/**28** 3:7) (**d**) halogenated derivatives, K_2_CO_3_, rt, 15–48 h or reflux, 3 h, 32–98% (**e**) LDA 1.0 M sol., dry THF, −78 °C then *tert*-butyl 4-(iodomethyl)piperidine-1-carboxylate, 1 h, −78 °C, 21–47% (**f**) TFA, CH_2_Cl_2_, rt, 1 h (**g**) bromomethylcyclohexane, K_2_CO_3_, DMF, 110 °C, 3 h, 23–79% (2 steps) (**h**) HBr sol. (33 wt.% in AcOH), 50 °C, 3 h, 85%.

Our last synthesized chemical series consisted in the study of the influence of the C4 substitution by various alkoxyls, while maintaining a C–C bond in C3. The mixture of oximes **25a**–**25b** was prepared in quantitative yield (*Z*/*E* 1:4) using a condensation reaction between the dihydroxyacetophenone and hydroxylamine. After separation of the both isomers, the ketoxime **25b** was firstly reacted with PPh_3_ and DDQ in dry THF at room temperature for 1 h but we obtained the undesired 2-methyl-1,3-benzoxazol-4-ol **27**^37^. Its structure was confirmed by X-ray diffraction of obtained crystals (see Supporting Information), The formation of **27** seems to be explained by the Beckmann rearrangement, allowing oximes to rearrange into substituted amides. The ketoxime **25b** was finally transformed into the corresponding activated oxime **26** by treatment with acetic anhydride for 1 h in 83% yield^38^. The acetylated intermediate was cyclized in pyridine to form the mixture of 1,3- and 1,2-benzoxazole **27**–**28** (3:7). At this stage, we introduced various alkyl groups (cyclo-, linear or branched alkyls) or benzyl on the hydroxyl group of the latter mixture **27**–**28** to obtain 1,3-benzoxazole **29a**–**e** and 1,2-benzoxazole **30a**–**e**, which were separated. The desired compounds **32a**–**e** were synthesized, under the same conditions as previously described, in a four-step sequence through the lithiation reaction of methylated compounds **30a**–**e**, the subsequent addition of the (iodomethyl)piperidine chains, the deprotection of the Boc groups before alkylating the piperidines by a methylcyclohexyl group (Fig. 4). Additionally, the compound **33** was obtained by *O*-debenzylation of the derivative **32e** using hydrobromic acid in acetic acid in 85% yield.

### *In vitro* evaluation

**Figure 5:**
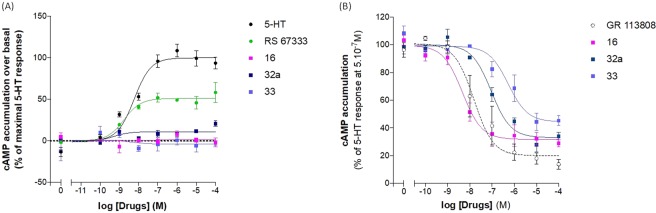
Determination of the 5-HT_4_ pharmacological profiles for compounds **16**, **32a** and **33** by quantification of cAMP production. (**A**) Assay to determine the agonism activity (with serotonin and the partial agonist RS-67333 as references). (**B**) Assay to determine the antagonism activity (with the antagonist GR113808 as reference). Data presented are the mean of three independent experiments performed in duplicate.

Compounds **11**–**16**, **21**–**23**, **32a**–**e** and **33** were evaluated *in vitro* for their capacity to inhibit *h*AChE using the spectrometric Ellman method^39^ and to bind to human 5-HT_4_R (*h*5-HT_4_R) in competition studies^40^ with the tritiated ligand [^3^H]–GR113808, a specific and highly potent 5-HT_4_R antagonist (Table 1). The 5-HT_4_R pharmacological profile was also determined for some compounds by quantification of cAMP production (Fig. 5).

**Table 1:** *h*AChE inhibitory activity and *h*5-HT_4_R affinity for benzisoxazole derivatives **11**–**16**, **21**–**23**, **32a**–**e** and **33**.

Compound				IC_50_ *h*AChE (nM) % inhibition at 10^−6^ M	*K*_i_ *h*5-HT_4_R (nM) % inhibition at 10^−6^ M/10^−8^ M
	X	R_1_	R_2_		
	donecopride			16 ± 5 (n = 2)	8.5 ± 0.3 (n = 3)
**11**	O	H		n.d. 24%	9.6 ± 1.6 (n = 3) 100%/67%
**12**	O	H		n.d. 33%	42.4 ± 12.2 (n = 3) 99%/29%
**13**	O	H		n.d. 8%	20.8 ± 12.5 (n = 3) 100%/59%
**14**	O			441 ± 63 (n = 2) 82%	2.7 ± 0.4 (n = 3) 100%/99%
**15**	O			939 ± 40 (n = 2) 82%	3.0 ± 0.4 (n = 3) 100%/94%
**16**	O			n.d. 23%	4.1 ± 0.4 (n = 3) 100%/100%
**21**	CH_2_	H		240 ± 59 (n = 2) 84%	139 ± 9 (n = 3) 89%/4%
**22**	CH_2_	H		35.7 ± 9.4 (n = 2) 96%	263 ± 11 (n = 3) 78%/8%
**23**	CH_2_	H		n.d. 11%	n.d. 87%/8%
**32a**	CH_2_			63.5 ± 19.2 (n = 2) 94%	59 ± 8.5 (n = 3) 100%/13%
**32b**	CH_2_			200 ± 32 (n = 2) 87%	80.5 ± 9.9 (n = 3) 100%/16%
**32c**	CH_2_			n.d. 42%	219 ± 65 (n = 3) 100%/4%
**32d**	CH_2_			1001 ± 84 (n = 2) 52%	616 ± 67 (n = 3) 100%/0%
**32e**	CH_2_			n.d. 10%	2920 ± 781 (n = 3) 96%/0%
**33**	CH_2_	OH		97.3 ± 1.5 (n = 2) 87%	37 ± 10.5 (n = 3) 100%/29%

## Discussion

Correlating well with the results reported by Brodney *et al*.^29^ the first unsubstituted 1,2-benzoxazoles **11**–**13** with an ether link displayed potent *in vitro* affinity towards 5-HT_4_R (similar to donecopride with a *K*_i_ of 9.6 nM for the benzisoxazole analogue **11**) but showed a loss of inhibition of the enzyme (Table 1).

Villalobos *et al*. have reported that, in comparison with unsubstituted compounds, an increase of AChE inhibition was observed by substitution of the benzisoxazole ring with electron-donating and some electron-withdrawing groups^27^. In accordance with these comments and in order to design donecopride-inspired compounds, the substitution of the benzisoxazole ring by a methoxy group in position C4 led to compounds **14** and **15** presenting a slight activity with a range of submicromolar inhibition of AChE (respectively 441 nM and 939 nM). The isobutyl-substituted piperidine derivative **16** did not show any inhibition of the enzyme. This isobutyl group probably cannot interact with AChE *via* the hydrophobic interaction conventionally observed with the tryptophan residue in the anionic subsite of the active site (residue Trp86 for *h*AChE). The introduction of the methoxy group also increased the *in vitro* capacity of derivatives **14**–**16** to bind to human 5-HT_4_R with an affinity of less than 5 nM.

Thanks to the pharmacomodulation of donecopride, we demonstrated that the modulation of the linker chain between the aromatic ring and the piperidine has an important impact on both pharmacological activities. Indeed, an amide or ester bond causes loss of AChE inhibition, while improving the affinity for 5-HT_4_R in comparison with two-carbon methylene bridge. Docking work has revealed a difference in the interaction of an amide or ester compound with the enzyme due to the rigidity induced by these structures (loss of one of the interactions)^41^.

The synthesis of compounds **21–22**, with a two-carbon methylene link, seems to confirm this hypothesis because the compounds showed, in comparison with the ether analogues **11–13**, much better AChE inhibition activity with respectively IC_50_s of 240 nM and 36 nM. The modification of an ether bond between the benzisoxazole and piperidine rings to a two-carbon methylene bond allows to increase flexibility and freedom of rotation of our structures, and to implement an AChE inhibition property. However, these structures presented in return a decrease in affinity towards 5-HT_4_R.

**Figure 6:**
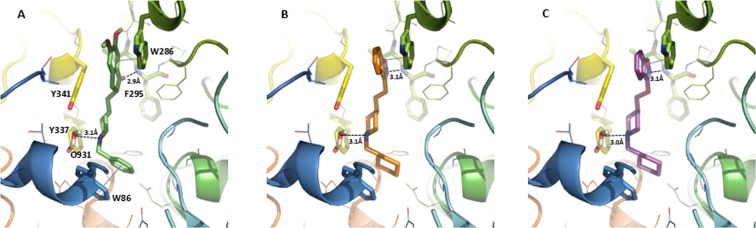
(**A**) Donepezil co-crystallized in the hAChE (PDB ED: 4EY7). Docking poses of the compound **21** (**B**) and the compound **11** (**C**) in *h*AChE binding sites. The compounds and the side chains of the binding site residues are in stick and the protein in ribbon representation. This figure was made with PYMOL (DeLano Scientific, 2002, San Carlo, USA).

In order to better understand this difference docking studies of compounds **11** and **21** were performed into a human AChE structure co-crystallised with donepezil (PDB ED: 4EY7^42^, Fig. 6A) using the GOLD software^43,44^. Firstly, compound **11** and **21** were built using Discovery Studio^45^ and protonated on piperidine ring following the ChemAxon software prediction (http://www.chemaxon.com/).

During the docking of compound **11** and **21** into human AChE a water molecule interacting with protonated piperidine ring of donepezil was conserved (residue number 931). In the selected best scoring pose for compound **21** (Fig. 6B, ChemPLP Score = 101.62) its orientation was closed to the donepezil one. Indeed, compound 21 reproduced well the donepezil interactions: (i) the charged nitrogen of the piperidine ring is oriented in a position suitable for interacting with the water molecule slotting between Tyr337 and Tyr341, (ii) the nitrogen atom of benzisoxazole ring was close to NH of the Phe295 backbone and formed with it a hydrogen bond and in the same time the benzisoxazole ring was positioned in a parallel way with respect to the Trp286 indole ring of the PAS at a 3.1 Å distance to favor the π-stacking interaction. (iii) the cyclohexane ring occupied the donepezil benzyl ring place in neighboring of Trp86 and when the cyclohexane ring was replaced by benzyl one (compound **22**) the AChE inhibition activities increased got closer to the donepezil one.

However, the best docked pose of compound **11** (ChemPLP Score = 98.21) does not allow us to lighten its worst AChE activity compared to the compound **21**. The three attachment points observed for compound **21** were well reproduced in the compound **11** docking pose (see Fig. 6C). We could however postulate that the replacement of the methylene linker present in compound **21** by an oxygen atom in compound **11** could increase its rigidity due to its mesomeric effect as earlier described in Donecopride analog^41^. This rigidity might affect its binding within human AChE binding site.

Finally, the last chemical series was synthesized with both the two-carbon methylene link and various alkoxyls in position C4. The compounds **32a–32e** demonstrated in most of the cases a correlation between the steric effect of the alkyl group and the results of both pharmacological activities. Except for the compound **32c** without AChE inhibition, the increasing bulk of the alkoxyl group (*O*-benzyl **32e** > *O*-cyclopentyl **32d** > *O*-isobutyl **32c** > *O*-ethyl **32b** > O-methyl **32a**) results in less % 5-HT_4_R affinity and AChE inhibition are interesting. The methoxy **32a** and the alcohol **33** were the most promising of all benzisoxazoles synthesized in this work, with respectively IC_50_ values of 63.5 nM and 97.3 nM towards AChE and *K*_i_ values of 59 nM and 37 nM for the 5-HT_4_R.

On the basis of these results, the two benzisoxazoles **32a** and **33** were selected to determine the 5-HT_4_R pharmacological profile of our compounds. In these assays both behaved as antagonists (Fig. 5) with IC_50_ of 97.2 ± 17.2 nM and 883.0 ± 597.8 nM, respectively. This profile is not in accordance with our objective. We determined also the 5-HT_4_R pharmacological profile of the compound **16** with an ether link and the compound also behaved as a highly potent antagonist (IC_50_ of 5.6 ± 2.2 nM).

**Figure 7:**
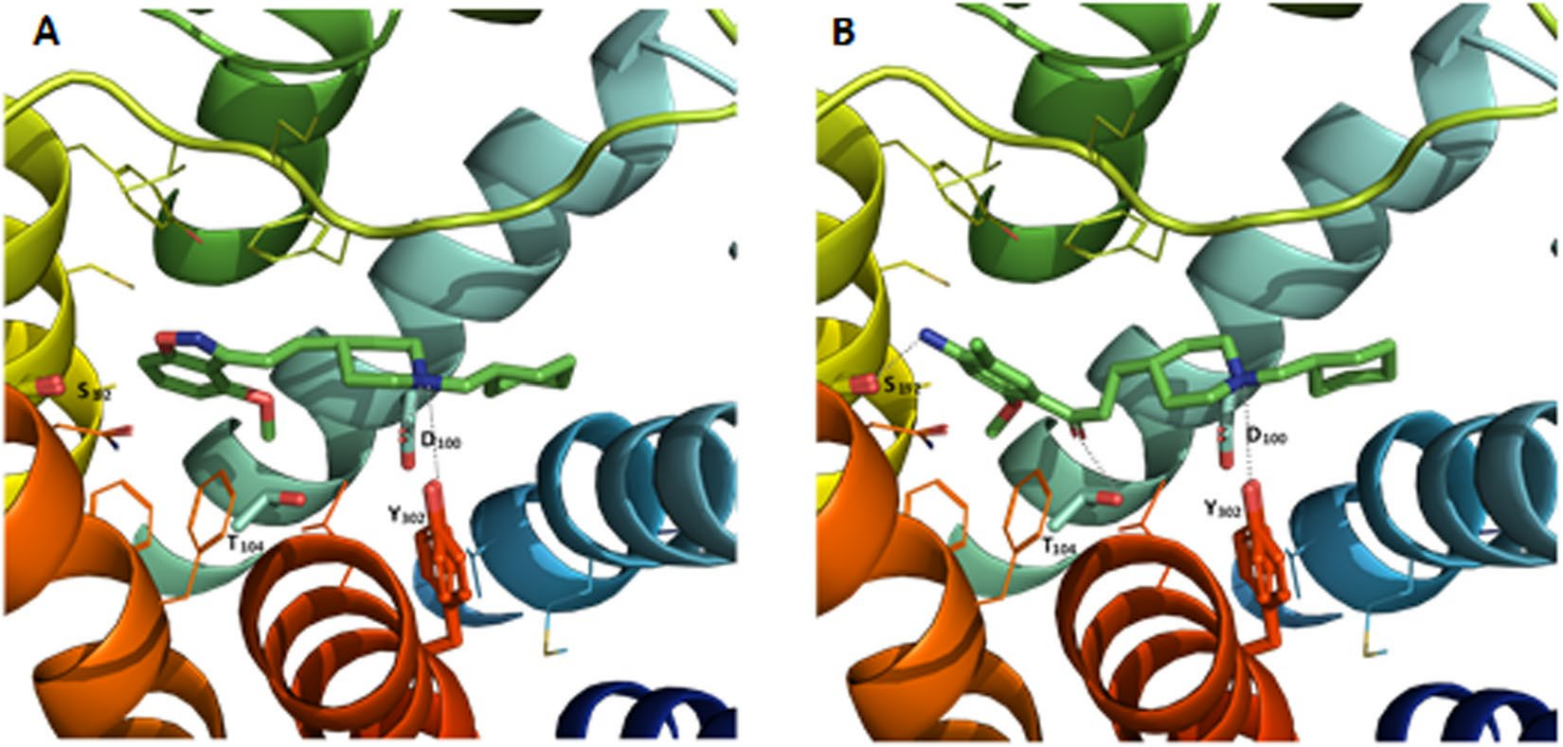
Compound **32a** (**A**) and Donecopride (**B**) positioned in the 5-HT_4_R binding sites from docking studies. The compound and the side chains of the binding site residues are in stick and the protein in ribbon representation. This figure was made with PYMOL (DeLano Scientific, 2002, San Carlo, USA).

In order to better understand their profile, docking studies were performed in a homology model of the 5-HT_4_ receptor. The docking of compound **32a** was compared to Donecopride^41^, and appear similar which could explain their affinities for the 5-HT_4_R (Fig. 7). For both compounds the basic piperidine nitrogen interacts with Asp100 on the transmembrane helix 3 (consistent with the constraint used during docking) and the aromatic rings are oriented towards the transmembrane helix 5 (TM5) (in yellow in Fig. 7). However two differences can be underlined between both compounds poses: (i) lack of an H-bond interaction for compound **32a** with Ser197 and (ii) lack of an electrostatic interaction for compound **32a** with Thr104. One of these two interactions could therefore be responsible for 5-HT_4_R agonism/antogonism profile.

On the basis of this docking study, we could postulate that further modulation of the benzisoxazole moiety, either by introducing novel substituent or by modulation of the methoxy group will be needed to obtain a 5-HT_4_R agonist. Such modulation should reproduce one or the two missing H-bonds with Ser197 or Thr104 to reproduce the agonist profile of Donecopride^41^ or compound [**A**]^29^.

## Conclusion

To briefly summarize, all benzisoxazoles synthesized allowed to establish structure-activity relationships and to study the impact of a pharmacomodulation of each part of the structure on the pharmacological results. In general, compounds with an ether link promote the affinity for 5-HT_4_R, whereas compounds with a two-carbon methylene link promote the inhibition of AChE. Several compounds have shown good activities *in vitro* on both targets and constitute a real basis for further works, in particular the compounds **32a** and **33**. It is necessary to combine all pharmacomodulations that have been performed to achieve the design of compounds meeting the selected criteria to belong to this MTDL family.

## Methods

### Chemistry

All commercially available compounds were used without further purification. Melting points were determined on a Köfler apparatus. Analytical thin-layer chromatography (TLC) was performed on silica gel 60 F_254_ on aluminium plates (Merck) and visualized with UV light (254 nm). Flash chromatography was conducted on a VWR SPOT II Essential instrument with silica gel 60 (40–63 µm). Column’s size and flow rate were used according to manufacturer’s recommendation. NMR spectra were recorded at 400 or 500 MHz (Bruker Avance III 400/500 MHz) for ^1^H NMR and at 100 or 125 MHz for ^13^C NMR in chloroform-*d*, methanol-*d*_4_ or DMSO-*d*_6_ with chemical shift (*δ*) given in parts per million (ppm) relative to TMS as internal standard and recorded at 295 K. The following abbreviations are used to describe peak splitting patterns when appropriate: br = broad, s = singlet, d = doublet, t = triplet, q = quartet, m = multiplet, dd = doublet of doublet, dt = doublet of triplet. Coupling constants *J* are reported in hertz units (Hz). Infrared spectra (IR) were obtained on a PERKIN-ELMER FT-IR spectrometer and are reported in terms of frequency of absorption (cm^−1^) using KBr discs. High-resolution mass spectra (HRMS) were obtained by electronic impact (HRMS/EI), or by electrospray (HRMS/ESI) on a Bruker maXis mass spectrometer. LC-MS (ESI) analyses were realized with Waters Alliance 2695 as separating module using the following gradients: A (95%)/B (5%) to A (5%)/B (95%) in 4.00 min. This ratio was hold during 1.50 min before return to initial conditions in 0.50 min. Initial conditions were then maintained for 2.00 min (A = H_2_O, B = CH_3_CN; each containing HCOOH: 0.1%; column XBridge C18 2.5 µm/4.6 × 50 mm; flow rate 0.8 mL/min). MS were obtained on a SQ detector by positive ESI. Mass spectrum data are reported as m/z. X-ray diffraction experiments were performed with graphite–monochromatized Mo Kα radiation on a Bruker-Nonius Kappa CCD area detector diffractometer either at 298 K or at 150 K IUPAC nomenclature was used for all compounds. For the description of NMR spectra, the numbering used for hydrogens can be different than the IUPAC numbering. Compounds, that are not fully characterized, may already have been described in the literature, according to the references cited. Yields refer to chromatographically and spectroscopically (^1^H NMR) homogeneous materials and are mostly unoptimized. NMR spectroscopy (^1^H) was used to determine the proportions of mixture^41^.

### Representative procedure for the synthesis of 11–16, 21–23 and 32a-e

To a stirred solution of *tert*-butyl piperidine-1-carboxylate derivative (1.0 eq.) in CH_2_Cl_2_ (20 mL/mmol) was added TFA (2 mL/mmol). The resulting mixture was stirred at room temperature for 1 h. Removal of the solvent under vacuum afforded the crude product, which was directly engaged in the next step. The residue obtained (1.0 eq.) was dissolved in the appropriate solvent (MeOH, DCM or EtOH, 10 mL/mmol), an alkyl-halogenated derivative (1.1 eq.) and a base (2.0–10.0 eq.) were then added. The resulting mixture was stirred at the appropriate temperature (room temperature, 80 °C or 110 °C) for 3–48 h, then concentrated *in vacuo*. Ethyl acetate was added, the organic layer was washed several times with brine, dried over MgSO_4_ and concentrated *in vacuo*. The crude was purified by chromatography on silica gel column and concentrated under reduced pressure to afford the corresponding alkylated compound^41^.

### 3-[[1-(cyclohexylmethyl)-4-piperidyl]methoxy]-1,2-benzoxazole (11)

The compound was prepared from *tert*-butyl 4-(1,2-benzoxazol-3-yloxymethyl)piperidine-1-carboxylate **9** (100 mg, 0.30 mmol, 1.0 eq.), bromomethylcyclohexane (47 µL, 0.33 mmol, 1.1 eq.) and K_2_CO_3_ (415 mg, 3.0 mmol, 10.0 eq.) in DMF according to the representative procedure **(E)** and stirring the reaction for 3 h at 110 °C. The crude was purified by flash chromatography on silica gel (cyclohexane/EtOAc, gradient 100:0 to 80:20) to give **11** as a yellow oil (22 mg, 22% yield over two steps); ^1^H NMR (CDCl_3_-*d*, 400 MHz) *δ* 7.65 (dt, ^3^*J* = 7.9 Hz, ^4^*J* = ^5^*J* = 1.0 Hz, 1H), 7.52 (ddd, ^3^*J* = 8.4 Hz, ^3^*J* = 7.0 Hz, ^4^*J* = 1.2 Hz, 1H), 7.43 (dt, ^3^*J* = 8.5 Hz, ^4^*J* = ^5^*J* = 0.8 Hz, 1H), 7.26 (m, 1H), 4.29 (d, ^3^*J* = 6.6 Hz, 2H), 2.92 (m, 2H), 2.12 (d, ^3^*J* = 7.0 Hz, 2H), 1.94–1.89 (m, 3H), 1.84–1.62 (m, 7H), 1.50–1.44 (m, 3H), 1.27–1.14 (m, 3H), 0.88 (m, 2H); ^13^C NMR (CDCl_3_-*d*, 100 MHz) *δ* 166.7, 163.9, 130.4, 122.9, 120.9, 114.4, 110.2, 74.8, 66.2, 53.9 (2 C), 35.8, 35.3, 32.1 (2 C), 28.8 (2 C), 26.8, 26.2 (2 C); LC-MS (ESI) t_R_ = 3.65 min; *m/z* [M + H]^+^ 329.59; HRMS/ESI: *m/z* calcd. for C_20_H_29_N_2_O_2_ [M + H]^+^ 329.2224, found 329.2224; IR (neat, cm^−1^) *ν* 2925, 2848, 2801, 2763, 1614, 1538, 1449, 1361, 1237, 1158, 989, 922, 751.

### Docking studies (hAChE)

For each docked compound a preliminary calculation on its protonation state at pH 7.4 was carried out using standard tools of the ChemAxon Package (Marvin 16.1.4.0, 2016, ChemAxon (http://www.chemaxon.com/) and the majority protonated microspecies at this pH was built by Discovery Studio software (Discovery Studio Modeling Environment, release 3.5, San Diego, CA: 2012) for docking studies.

The crystallographic coordinates of human acetylcholinesterase used for docking studies were obtained from X-ray structure of the donepezil/AChE complex (PDB ID 4EY7, a structure refined to 2.35 Å with an R factor of 17.7%)^41^.

The docking of the compounds into the hAChE was carried out with the GOLD program (v5.6.3) using the default parameters^43,44^. This program applies a genetic algorithm to explore conformational spaces and ligand binding modes. To evaluate the proposed ligand positions, the ChemPLP fitness function was applied in these docking studies. The binding site in the hAChE model was defined as a 7 Å sphere from the co-crystallized ligand donepezil and a water molecule interacting with protonated piperidine ring of donepezil was conserved during the docking (residue number 931).

### Docking studies (5-HT_4_R)

In this study, the 3D model of the human 5-HT4R built previously by homology sequence approach^46^ was used. This model was generated using the crystal structure (PBD: 2RH1) of the human β2 adrenergic receptor-T4 lysozyme fusion protein complexed with the carazolol^47^ as a template. The docking of the **32a** compound and donecopride into the generated model was carried out with the GOLD program (v5.7.3) using the default parameters^43,44^ and the proposed ligand positions were evaluated by the ChemPLP fitness function. The binding site in the 5-HT_4_R model was defined as a 10 Å sphere centred on the aspartic acid residue Asp100. As the mutagenesis studies have shown that the interaction between the positively ionisable amine of ligands and Asp100 of 5-HT_4_R is crucial for ligand binding, a hydrogen bond constraint between positively ionisable amine ligand and OD atom of Asp100 was used during the docking^48^. Furthermore, special attention was paid during the docking procedure to the following amino acids in the binding site, which were kept flexible: Arg96, Asp100, Thr104, Tyr192, Ser197 and Trp294.

### Pharmacological characterization of drugs on human 5-HT_4_R

The method was validated from saturation studies: six concentrations of [^3^H]GR113808 were used to give final concentrations of 0.0625–2 nM, and nonspecific binding of [^3^H]GR113808 was defined in the presence of 30 μM serotonin to determine the K_d_ and the B_max_. The competition studies used membrane preparations made from proprietary stable recombinant cell lines expressing the 5-HT_4(b)_ receptor to ensure high-level of GPCR surface expression (HTS110M, Millipore). Membranes (2.5 µg protein) were incubated in duplicate at 25 °C for 60 min in the absence or the presence of 10^–6^ or 10^–8^M of each drug and 0.2 nM [^3^H]-GR 113808 (VT 240, ViTrax) in 25 mM Tris buffer (pH 7.4, 25 °C). At the end of the incubation, homogenates were filtered through Whatman GF/C filters (Alpha Biotech) pre-soaked with 0.5% polyethylenimine using a Brandel cell harvester. Filters were subsequently washed three times with 4 mL of ice-cold 25 mM Tris buffer (pH 7.4, 4 °C). Non-specific binding was evaluated in parallel in the presence of 30 μM serotonin.

For some of these compounds, affinity constants were calculated from five-point inhibition curves using the EBDA-Ligand software and expressed as Ki ± SD^41^.

### *In vitro* tests of AChE biological activity

Inhibitory capacity of compounds on AChE biological activity was evaluated through the use of the spectrometric method of Ellman^39^. Acetylthiocholine and 5,5-dithiobis-(2-nitrobenzoic) acid (DTNB) were purchased from Sigma Aldrich. AChE from human erythrocytes (buffered aqueous solution, ≥500 units/mg protein (BCA), Sigma Aldrich) was diluted in 20 mM HEPES buffer pH 8, 0.1% Triton X-100 such as to have enzyme solution with 0.25 unit/mL enzyme activity. In the procedure, 100 μL of 0.3 mM DTNB dissolved in phosphate buffer pH 7.4 were added into the 96 wells plate followed by 50 μL of test compound solution and 50 μL of enzyme (0.05 U final). After 5 min of preincubation at 25 °C, the reaction was then initiated by the injection of 50 μL of 10 mM acetylthiocholine iodide solution. The hydrolysis of acetylthiocholine was monitored by the formation of yellow 5-thio-2-nitrobenzoate anion as the result of the reaction of DTNB with thiocholine, released by the enzymatic hydrolysis of acetylthiocholine, at a wavelength of 412 nm using a 96-well microplate plate reader (TECAN Infinite M200, Lyon, France). Test compounds were dissolved in analytical grade DMSO. Donepezil was used as a reference standard. The rate of absorbance increase at 412 nm was followed every minute for 10 min. Assays were performed with a blank containing all components except acetylthiocholine, in order to account for non-enzymatic reaction. The reaction slopes were compared and the percent inhibition due to the presence of test compounds was calculated by the following expression: 100 − (vi/v0 × 100) where vi is the rate calculated in the presence of inhibitor and v0 is the enzyme activity.

First screening of AChE activity was carried out at a 10^−6^ or 10^−5^ M concentration of compounds under study. For the compounds with significant inhibition (≥50%), IC_50_ values were determined graphically by plotting the % inhibition versus the logarithm of six inhibitor concentrations in the assay solution using the Origin software.

### Determination of cAMP production

COS-7 cells were purchased from ATCC (ATCC CRL-1651; LGC STANDARTS, Molsheim, France). T were grown in Dulbecco’s modified Eagle medium (DMEM) supplemented with 10% dialyzed fetal calf serum (dFCS) and antibiotics. Cells were transiently transfected with plasmid encoding HA-tagged human 5-HT_4_ receptor, then seeded in 96-well plates (35,000 cells/well). 24 hrs after transfection, cells were washed once with 200 µl of HBS (20 mM HEPES; 150 mM NaCl; 4.2 mM KCl; 0.9 mM CaCl_2_; 0.5 mM MgCl_2_; 0.1% glucose; 0.1% BSA) and, after HBS removal, exposed to the indicated concentrations of 5-HT_4_R ligands in the presence of 0.1 mM of the phosphodiesterase inhibitor RO-20-1724, at 37 °C in 100 µl of HBS. After 10 min, cells were then lysed by addition of the same volume of Triton-X100 (0.1%) during 30 min at 37 °C. The competition assay in antagonist mode was performed as follows: after one wash in 200 µl of HBS, cells were exposed to 5-HT_4_R ligands at twice the indicated concentration, at 37 °C in 50 µl of HBS. After 7 min, serotonin (final concentration of 5.10^−7^M) was added in 50 µl of HBS in the presence of the phosphodiesterase inhibitor RO-20-1724 (final concentration of 0.1 mM). After 10 min, cells were then lysed by addition of the same volume of Triton-X100 (0.1%) during 30 min at 37 °C. Quantification of cAMP production was performed by HTRF by using the cAMP Dynamic kit (Cisbio Bioassays) according to the manufacturer’s instructions

## Supplementary information

Supplementary Data.
