# Supplementary material for: Rational design of novel benzisoxazole derivatives with acetylcholinesterase inhibitory and serotoninergic 5-HT4 receptors activities for the treatment of Alzheimer’s disease
Source: Sci Rep. 2020 Feb 20;10:3014. doi: 10.1038/s41598-020-59805-7 (PMC7033111; doi:10.1038/s41598-020-59805-7)
Supplement: Supplementary file 1 — Supplementary Data. [file 41598_2020_59805_MOESM1_ESM.docx]

**Rational design of novel benzisoxazole derivatives with acetylcholinesterase inhibitory and serotoninergic 5-HT4 receptor activities for the treatment of Alzheimer's disease** **(Supplementary Information)**

## Julien Lalut,^1^ Hugo Payan,^2^ Audrey Davis,^1^ Cédric Lecoutey,^1^ Rémi Legay,^1^ Jana Sopkova-de Oliveira Santos,^1^ Sylvie Claeysen,^2^ Patrick Dallemagne^1^ & Christophe Rochais^1^

### ^1^Centre d’Etudes et de Recherche sur le Médicament de Normandie, Normandie Univ, UNICAEN, CERMN, 14000 Caen, France.

### ^2^ IGF, Univ. Montpellier, CNRS, INSERM, Montpellier, France.

^*^Corresponding Author: christophe.rochais@unicaen.fr

**Contents of Supplementary Information**

**General experimental conditions - S1 -**

**General representative procedures (A)-(H) - S2 -**

**Experimental protocols - S3 -**

**Crystallographic data of compounds 7, 25b, 27, 30a and 30e - S4 -**

**References - S5 -**

**General experimental conditions**

**Chemistry.** All commercially available compounds were used without further purification. Melting points were determined on a Köfler apparatus. Analytical thin-layer chromatography (TLC) was performed on silica gel 60 F_254_ on aluminium plates (Merck) and visualized with UV light (254 nm). Flash chromatography was conducted on a VWR SPOT II Essential instrument with silica gel 60 (40-63µm). Column’s size and flow rate were used according to manufacturer’s recommendation. NMR spectra were recorded at 400 or 500 MHz (Bruker Avance III 400/500 MHz) for ^1^H NMR and at 100 or 125 MHz for ^13^C NMR in chloroform-*d*, methanol-*d_4_* or DMSO-*d_6_* with chemical shift (*δ*) given in parts per million (ppm) relative to TMS as internal standard and recorded at 295K. The following abbreviations are used to describe peak splitting patterns when appropriate: br = broad, s = singlet, d = doublet, t = triplet, q = quartet, m = multiplet, dd = doublet of doublet, dt = doublet of triplet. Coupling constants *J* are reported in hertz units (Hz). Infrared spectra (IR) were obtained on a PERKIN-ELMER FT-IR spectrometer and are reported in terms of frequency of absorption (cm^-1^) using KBr discs. High-resolution mass spectra (HRMS) were obtained by electronic impact (HRMS/EI), or by electrospray (HRMS/ESI) on a Bruker maXis mass spectrometer. LC-MS (ESI) analyses were realized with Waters Alliance 2695 as separating module using the following gradients: A (95%)/B (5%) to A (5%)/B (95%) in 4.00min. This ratio was hold during 1.50 min before return to initial conditions in 0.50 min. Initial conditions were then maintained for 2.00 min (A = H_2_O, B = CH_3_CN; each containing HCOOH: 0.1 %; column XBridge C18 2.5 µm/4.6x50 mm; flow rate 0.8 mL/min). MS were obtained on a SQ detector by positive ESI. Mass spectrum data are reported as m/z. X-ray diffraction experiments were performed with graphite–monochromatized Mo Kα radiation on a Bruker-Nonius Kappa CCD area detector diffractometer either at 298K or at 150K. Program used to solve structure: *SHELXS97*.**^[^^[[1]](#footnote-1)]^** Program used to refine structure: *SHELXL97*.**^[^^[[2]](#footnote-2)]^** IUPAC nomenclature was used for all compounds. For the description of NMR spectra, the numbering used for hydrogens can be different than the IUPAC numbering. Compounds, that are not fully characterized, may already have been described in the literature, according to the references cited. Yields refer to chromatographically and spectroscopically (^1^H NMR) homogeneous materials and are mostly unoptimized. NMR spectroscopy (^1^H) was used to determine the proportions of mixture.

**General representative procedures (A)-(H)**

**Representative procedure (A) for the synthesis of 3 and 4**. To a stirred solution of 2-hydroxybenzoic acid derivative (1.0 eq.) in MeOH (4 mL/mmol) was added dropwise concentrated H_2_SO_4_ (0.22 mL/mmol) and the resulting mixture was refluxed for 48 h. After cooling to room temperature, the mixture was concentrated *in vacuo*. The residue was dissolved with H_2_O, neutralized with NaHCO_3_ and extracted several times with EtOAc. The combined organic extracts were washed with brine, dried over MgSO_4_ and concentrated under reduced pressure to afford the corresponding methyl ester derivative.

**Representative procedure (B) for the synthesis of 5 and 6**. To a stirred solution of hydroxylamine hydrochloride (1.5 eq.) in H_2_O (1.5 mL/mmol) was added NaOH (3.5 eq.) and the resulting mixture was stirred at room temperature for 1 h under N_2_ atmosphere. Methyl ester derivative (1.0 eq.) in dioxane (500 µL/mmol) was added and the mixture was stirred at 40 °C for 15 h. After cooling to room temperature, the mixture was concentrated *in vacuo*, was made acid by addition of 1 N HCl solution and extracted several times with EtOAc. The combined organic extracts were dried over MgSO_4_ and concentrated *in vacuo*. The crude was purified by chromatography on silica gel column and concentrated under reduced pressure to afford the corresponding hydroxamic acid derivative.

**Representative procedure (C) for the synthesis of 7 and 8**. To a stirred solution of hydroxamic acid derivative (1.0 eq.) in dry THF (5 mL/mmol) was added a solution of CDI (2.0 eq.) in dry THF (5 mL/mmol) under N_2_ atmosphere. The resulting mixture was refluxed for 3 h. After cooling to room temperature, the mixture was concentrated *in vacuo*. The crude was dissolved with H_2_O, then was precipitated by addition of 6 N HCl solution and collected by filtration to afford the corresponding benzoxazole derivative.

**Representative procedure (D) for the synthesis of 9 and 10**. To a stirred solution of benzoxazole derivative (1.0 eq.) in dry THF (7 mL/mmol) was added *tert*-butyl 4-(hydroxymethyl)piperidine-1-carboxylate (1.2 eq.), PPh_3_ (2.0 eq.) and DEAD (2.0 eq.). The resulting mixture was refluxed for 19 h. After cooling to room temperature, the mixture was concentrated *in vacuo*. The crude was dissolved with EtOAc, then the organic layer was washed with brine, dried over MgSO_4_ and concentrated *in vacuo*. The crude was purified by chromatography on silica gel column and concentrated under reduced pressure to afford the corresponding Mitsunobu reaction product.

**Representative procedure (E) for the synthesis of 11-16, 21-23 and 32a-e**. To a stirred solution of *tert*-butyl piperidine-1-carboxylate derivative (1.0 eq.) in CH_2_Cl_2_ (20 mL/mmol) was added TFA (2 mL/mmol). The resulting mixture was stirred at room temperature for 1 h. Removal of the solvent under vacuum afforded the crude product, which was directly engaged in the next step. The residue obtained (1.0 eq.) was dissolved in the appropriate solvent (MeOH, DCM or EtOH, 10 mL/mmol), an alkyl-halogenated derivative (1.1 eq.) and a base (2.0–10.0 eq.) were then added. The resulting mixture was stirred at the appropriate temperature (room temperature, 80 °C or 110 °C) for 3–48 h, then concentrated *in vacuo*. Ethyl acetate was added, the organic layer was washed several times with brine, dried over MgSO_4_ and concentrated *in vacuo*. The crude was purified by chromatography on silica gel column and concentrated under reduced pressure to afford the corresponding alkylated compound.

**Representative procedure (F) for the synthesis of 18a-b and 25a-b.** To a stirred solution of hydroxylamine hydrochloride (1.5 eq.) in H_2_O (4 mL/mmol) was added NaOAc (4.0 eq.) and the mixture was stirred at room temperature for 1 h. The acetophenone derivative (1.0 eq.) in EtOH (5 mL/mmol) were added and the mixture was refluxed for 2–3 h. After cooling to room temperature, the mixture was concentrated *in vacuo*, was acidified with 1 N HCl solution and extracted several times with EtOAc. The combined organic extracts were washed with brine, dried over MgSO_4_ and then concentrated *in vacuo.* The residue was purified by chromatography on silica gel column and concentrated under reduced pressure to afford the corresponding oxime derivative.

**Representative procedure (G) for the synthesis of 20 and 31a-e.** 1.0 M LDA solution (1.0 eq.–2.2 eq.) was added dropwise to a cold stirred solution of methyl-benzoxazol derivative (1.0 eq.) in dry THF (5 mL/mmol) at –78 °C. After addition was complete, a solution of *tert*-butyl 4-(iodomethyl)piperidine-1-carboxylate **(3)** (1.05 eq.) in dry THF (5 mL/mmol) was added all at once. The resulting mixture was stirred for 1 h at –78 °C. Saturated NH_4_Cl solution was added, and the mixture was extracted with EtOAc. The combined organic layer was washed with brine, dried over MgSO_4_ and concentrated *in vacuo*. The crude was purified by chromatography on silica gel column to afford expected compound**.**

**Representative procedure (H) for the synthesis of 29a-e and 30a-e.** To a stirred solution of the mixture of 2-methyl-1,3-benzoxazol-4-ol **27** and 3-methyl-1,2-benzoxazol-4-ol **28** (1.0 eq.) in DMF (2.5 mL/mmol) were added K_2_CO_3_ (4.0 eq.) and the halogenated compound (1.1 eq.). The resulting mixture was stirred at room temperature, at 80 °C or at 110 °C for 3–48 h and then concentrated *in vacuo*. The residue was dissolved with EtOAc, washed with brine, the organic layer was dried over MgSO_4_. The crude was purified by chromatography on silica gel column and concentrated under reduced pressure to afford the corresponding alkylated compound.

**Experimental protocols**

**Methyl 2-hydroxybenzoate (3).** The compound was prepared from 2-hydroxybenzoic acid **1** (10.0 g, 72.4 mmol, 1.0 eq.) according to the representative procedure **(A)** and obtained as a colorless oil (10.37 g, 94%); ^1^H NMR (CDCl_3_-*d*, 400 MHz) *δ* 10.77 (br s, 1H), 7.84 (dd, *^3^J* = 8.2 Hz, *^4^J* = 1.8 Hz, 1H), 7.46 (ddd, *^3^J* = 8.5 Hz, *^3^J* = 7.2 Hz, *^4^J* = 1.8 Hz, 1H), 6.98 (dd, *^3^J* = 8.5 Hz, *^4^J* = 1.0 Hz, 1H), 6.88 (ddd, *^3^J* = 8.2 Hz, *^3^J* = 7.3 Hz, *^4^J* = 1.1 Hz, 1H), 3.95 (s, 3H); ^13^C NMR (CDCl_3_-*d*, 100 MHz) *δ* 170.7, 161.7, 135.8, 130.0, 119.3, 117.7, 112.5, 52.4; IR (neat, cm^-1^) *ν* 3439–3289, 3013, 2925, 2858, 1649, 1449, 1253, 1034, 881, 755, 647. Spectroscopic data consistent with that reported in the literature.**^[^^[[3]](#footnote-3)]^**

**Methyl 2-hydroxy-6-methoxy-benzoate (4).** The compound was prepared from 2-hydroxy-6-methoxy-benzoic acid **2** (4.0 g, 23.8 mmol, 1.0 eq.) according to the representative procedure **(A)** and obtained as a white solid (3.97 g, 92%); mp 54 °C; ^1^H NMR (CDCl_3_-*d*, 400 MHz) *δ* 11.5 (br s, 1H), 7.33 (t, *^3^J* = 8.3 Hz, 1H), 6.60 (dd, *^3^J* = 8.4 Hz, *^4^J* = 1.0 Hz, 1H), 6.42 (dd, *^3^J* = 8.5 Hz, *^4^J* = 0.9 Hz, 1H), 3.95 (s, 3H), 3.86 (s, 3H); ^13^C NMR (CDCl_3_-*d*, 100 MHz) *δ* 171.8, 163.7, 161.0, 135.3, 110.1, 103.1, 102.3, 56.2, 52.5; IR (neat, cm^-1^) *ν* 3249, 3011, 2981, 2956, 2844, 1655, 1232, 1087, 811. Spectroscopic data consistent with that reported in the literature.**^[^^[[4]](#footnote-4)]^**

**2-hydroxybenzenecarbohydroxamic acid (5).** The compound was prepared from methyl 2-hydroxybenzoate **3** (4.60 g, 30.2 mmol, 1.0 eq.) according to the representative procedure **(B)** and obtained without chromatographic purification as a white solid (4.30 g, 93%); mp 179 °C; ^1^H NMR (MeOD-*d_4_*, 400 MHz) *δ* 7.65 (dd, *^3^J* = 8.2 Hz, *^4^J* = 1.6 Hz, 1H), 7.35 (ddd, *^3^J* = 8.6 Hz, *^3^J* = 7.2 Hz, *^4^J* = 1.7 Hz, 1H), 6.90 (dd, *^3^J* = 8.6 Hz, *^4^J* = 0.8 Hz, 1H), 6.87 (ddd, *^3^J* = 8.2 Hz, *^3^J* = 7.3 Hz, *^4^J* = 1.2 Hz, 1H); ^13^C NMR (MeOD-*d_4_*, 100 MHz) *δ* 168.7, 160.3, 134.6, 128.4, 120.2, 118.3, 115.4; IR (neat, cm^-1^) *ν* 3286–2293, 2866, 1615, 1251, 1025, 993, 896, 745. Spectroscopic data consistent with that reported in the literature.**^[^^[[5]](#footnote-5)]^**

**2-hydroxy-6-methoxy-benzenecarbohydroxamic acid (6).** The compound was prepared from methyl 2-hydroxy-6-methoxy-benzoate **4** (2.20 g, 12.1 mmol, 1.0 eq.) according to the representative procedure **(B)**. The crude was purified by flash chromatography on silica gel (cyclohexane/CH_2_Cl_2_, gradient 50:50 to 0:100) to give **6** as a white solid (1.44 g, 65% yield); mp 138 °C; ^1^H NMR (CDCl_3_-*d*, 400 MHz) *δ* 12.74 (br s, 1H), 10.49 (br s, 1H), 7.84 (br s, 1H), 7.31 (t, *^3^J* = 8.5 Hz, 1H), 6.65 (dd, *^3^J* = 8.6 Hz, *^4^J* = 0.9 Hz, 1H), 6.43 (dd, *^3^J* = 8.3 Hz, *^4^J* = 0.9 Hz, 1H), 3.96 (s, 3H); ^13^C NMR (CDCl_3_-*d*, 100 MHz) *δ* 166.5, 163.7, 158.4, 134.2, 112.0, 101.4, 101.3, 56.5; HRMS/ESI: *m/z* calcd. for C_8_H_10_NO_4_ [M+H]^+^ 184.0604, found 184.0603; IR (neat, cm^-1^) *ν* 3360, 3242, 3011–2571, 2952, 2851, 1627, 1588, 1464, 1244, 1091, 801.

**1,2-benzoxazol-3-ol (7).** The compound was prepared from 2-hydroxybenzenecarbohydroxamic acid **5** (8.5 g, 55.5 mmol, 1.0 eq.) according to the representative procedure **(C)** and obtained as a white solid (7.04 g, 94%); mp 138 °C; ^1^H NMR (CDCl_3_-*d*, 400 MHz) *δ* 7.80 (ddd, *^3^J* = 7.9 Hz, *^4^J* = 1.2 Hz, *^5^J* = 0.8 Hz, 1H), 7.60 (ddd, *^3^J* = 8.4 Hz, *^3^J* = 7.1 Hz, *^4^J* = 1.0 Hz, 1H), 7.43 (dt, *^3^J* = 8.5 Hz, *^4^J* = *^5^J* = 1.0 Hz, 1H), 7.32 (ddd, *^3^J* = 7.9 Hz, *^3^J* = 7.1 Hz, *^4^J* = 1.0 Hz, 1H); ^13^C NMR (CDCl_3_-*d*, 100 MHz) *δ* 166.4, 163.1, 131.9, 123.6, 122.4, 114.5, 110.4; HRMS/ESI: *m/z* calcd. for C_7_H_6_NO_2_ [M+H]^+^ 136.0393, found 136.0389; IR (neat, cm^-1^) *ν* 3101–2579, 2934, 2835, 1614, 1447, 1231, 752.

**4-methoxy-1,2-benzoxazol-3-one (8).** The compound was prepared from 2-hydroxy-6-methoxy-benzenecarbohydroxamic acid **6** (1.44 g, 7.9 mmol, 1.0 eq.) according to the representative procedure **(C)** and obtained in a mixture of 4-methoxy-1,2-benzoxazol-3-one **8**/4-methoxy-3*H*-1,3-benzoxazol-2-one **8’** (80:20) as a white solid (1.21 g, 93% yield) (895 mg, 74% yield for derivative **8**); HRMS/ESI: *m/z* calcd. for C_8_H_8_NO_3_ [M+H]^+^ 166.0499, found 166.0497; IR (neat, cm^-1^) *ν* 3174–2588, 2936, 2858, 1764, 1465, 1251, 1071, 770.

^1^H NMR (CDCl_3_-*d*, 400 MHz) *δ* 7.49 (t, ^3^*J* = 8.2 Hz, 1H), 6.97 (d, ^3^*J* = 8.4 Hz, 1H), 6.63 (d, ^3^*J* = 8.0 Hz, 1H), 5.34 (br s, 1H), 4.01 (s, 3H); ^13^C NMR (CDCl_3_-*d*, 100 MHz) *δ* 165.8, 165.3, 156.1, 133.6, 104.3, 103.6, 103.0, 56.2;

^1^H NMR (CDCl_3_-*d*, 400 MHz) *δ* 9.03 (br s, 1H), 7.05 (t, ^3^*J* = 8.3 Hz, 1H), 6.86 (d, ^3^*J* = 8.2 Hz, 1H), 6.73 (d, ^3^*J* = 8.4 Hz, 1H), 3.91 (s, 3H); ^13^C NMR (CDCl_3_-*d*, 100 MHz) *δ* 155.4, 144.8, 144.5, 123.0, 118.5, 106.8, 103.5, 56.1;

***Tert*-butyl 4-(1,2-benzoxazol-3-yloxymethyl)piperidine-1-carboxylate (9).** The compound was prepared from 1,2-benzoxazol-3-ol **7** (300 mg, 2.22 mmol, 1.0 eq.) according to the representative procedure **(D)**. The crude was purified by flash chromatography on silica gel (cyclohexane/EtOAc, gradient 100:0 to 80:20) to give **9** as a white solid (432 mg, 44% yield); mp 95 °C; ^1^H NMR (CDCl_3_-*d*, 400 MHz) *δ* 7.62 (ddd, *^3^J* = 7.9 Hz, *^4^J* = 1.2 Hz, *^5^J* = 0.8 Hz, 1H), 7.52 (ddd, *^3^J* = 8.3 Hz, *^3^J* = 7.0 Hz, *^4^J* = 1.2 Hz, 1H), 7.42 (dt, *^3^J* = 8.5 Hz, *^4^J* = *^5^J* = 0.8 Hz, 1H), 7.25 (ddd, *^3^J* = 7.9 Hz, *^3^J* = 7.1 Hz, *^4^J* = 0.8 Hz, 1H), 4.29 (d, *^3^J* = 6.6 Hz, 2H), 4.16 (m, 2H), 2.76 (m, 2H), 2.10 (m, 1H), 1.83 (m, 2H), 1.46 (s, 9H), 1.31 (m, 2H); ^13^C NMR (CDCl_3_-*d*, 100 MHz) *δ* 166.6, 164.0, 154.9, 130.5, 123.1, 120.9, 114.3, 110.3, 79.6, 74.4, 43.5 (2C), 35.9, 28.7 (2C), 28.6 (3C); HRMS/ESI: *m/z* calcd. for C_18_H_25_N_2_O_4_ [M+H]^+^ 333.1809, found 333.1806; IR (neat, cm^-1^) *ν* 2978, 2949, 2861, 1687, 1615, 1536, 1365, 1238, 1140, 753.

***Tert*-butyl 4-[(4-methoxy-1,2-benzoxazol-3-yl)oxymethyl]piperidine-1-carboxylate (10).** The compound was prepared from a mixture of compounds **8**/**8’** 80:20 (300 mg, 1.82 mmol, 1.0 eq.) according to the representative procedure **(D)**. The crude was purified by flash chromatography on silica gel (cyclohexane/EtOAc, gradient 100:0 to 90:10) to isolate **10** as a colorless oil (432 mg, 65% yield) after concentration *in vacuo* and then (cyclohexane/EtOAc, gradient 90:10 to 80:20) to isolate **10’** as a white solid (95 mg, 14% yield) after concentration *in vacuo*.

**Compound 10.** ^1^H NMR (CDCl_3_-*d*, 400 MHz) *δ* 7.42 (t, ^3^*J* = 8.2 Hz, 1H), 7.00 (d, ^3^*J* = 8.4 Hz, 1H), 6.59 (d, ^3^*J* = 8.0 Hz, 1H), 4.28 (d, ^3^*J* = 6.7 Hz, 2H), 4.16 (m, 2H), 3.94 (s, 3H), 2.77 (m, 2H), 2.14 (m, 1H), 1.85 (m, 2H), 1.46 (s, 9H), 1.30 (m, 2H_'_); ^13^C NMR (CDCl_3_-*d*, 100 MHz) *δ* 166.4, 166.1, 155.1, 155.0, 132.1, 104.3, 103.3, 103.0, 79.6, 74.5, 56.1, 43.5 (2C), 35.8, 28.7 (2C), 28.6 (3C); HRMS/ESI: *m/z* calcd. for C_19_H_27_N_2_O_5_ [M+H]^+^ 363.1914, found 363.1912; IR (neat, cm^-1^) *ν* 2975, 2936, 2855, 1691, 1613, 1429, 1365, 1102, 980, 785.

**Compound 10’.** mp 117 °C; ^1^H NMR (CDCl_3_-*d*, 400 MHz) *δ* 7.02 (t, ^3^*J* = 8.3 Hz, 1H), 6.83 (dd, ^3^*J* = 8.1 Hz, ^4^*J* = 0.8 Hz, 1H), 6.73 (dd, ^3^*J* = 8.5Hz, ^4^*J* = 0.7 Hz, 1H), 4.09 (m, 2H), 3.89 (s, 3H), 3.87 (d, ^3^*J* = 7.2Hz, 2H), 2.65 (m, 2H), 2.00 (m, 1H), 1.62 (m, 2H), 1.43 (s, 9H), 1.25 (m, 2H); ^13^C NMR (CDCl_3_-*d*, 100 MHz) *δ* 154.9, 154.8, 144.9, 143.6, 122.7, 119.6, 107.1, 103.5, 79.5, 56.1, 49.0, 43.5 (2C), 36.6, 29.4 (2C), 28.5 (3C); HRMS/ESI: *m/z* calcd. for C_19_H_27_N_2_O_5_ [M+H]^+^ 363.1914, found 363.1915; IR (neat, cm^-1^) *ν* 2974, 2935, 2854, 1774, 1690, 1470, 1253, 1173, 1061, 765.

**3-[[1-(cyclohexylmethyl)-4-piperidyl]methoxy]-1,2-benzoxazole (11).** The compound was prepared from *tert*-butyl 4-(1,2-benzoxazol-3-yloxymethyl)piperidine-1-carboxylate **9** (100 mg, 0.30 mmol, 1.0 eq.), bromomethylcyclohexane (47 µL, 0.33 mmol, 1.1 eq.) and K_2_CO_3_ (415 mg, 3.0 mmol, 10.0 eq.) in DMF according to the representative procedure **(E)** and stirring the reaction for 3 h at 110 °C. The crude was purified by flash chromatography on silica gel (cyclohexane/EtOAc, gradient 100:0 to 80:20) to give **11** as a yellow oil (22 mg, 22% yield over two steps); ^1^H NMR (CDCl_3_-*d*, 400 MHz) *δ* 7.65 (dt, *^3^J* = 7.9 Hz, *^4^J* = *^5^J* = 1.0 Hz, 1H), 7.52 (ddd, *^3^J* = 8.4 Hz, *^3^J* = 7.0 Hz, *^4^J* = 1.2 Hz, 1H), 7.43 (dt, *^3^J* = 8.5 Hz, *^4^J* = *^5^J* = 0.8 Hz, 1H), 7.26 (m, 1H), 4.29 (d, ^3^*J* = 6.6 Hz, 2H), 2.92 (m, 2H), 2.12 (d, ^3^*J* = 7.0 Hz, 2H), 1.94–1.89 (m, 3H), 1.84–1.62 (m, 7H), 1.50–1.44 (m, 3H), 1.27–1.14 (m, 3H), 0.88 (m, 2H); ^13^C NMR (CDCl_3_-*d*, 100 MHz) *δ* 166.7, 163.9, 130.4, 122.9, 120.9, 114.4, 110.2, 74.8, 66.2, 53.9 (2C), 35.8, 35.3, 32.1 (2C), 28.8 (2C), 26.8, 26.2 (2C); LC-MS (ESI) t_R_ = 3.65 min; *m/z* [M+H]^+^ 329.59; HRMS/ESI: *m/z* calcd. for C_20_H_29_N_2_O_2_ [M+H]^+^ 329.2224, found 329.2224; IR (neat, cm^-1^) *ν* 2925, 2848, 2801, 2763, 1614, 1538, 1449, 1361, 1237, 1158, 989, 922, 751.

**3-[(1-benzyl-4-piperidyl)methoxy]-1,2-benzoxazole (12).** The compound was prepared from *tert*-butyl 4-(1,2-benzoxazol-3-yloxymethyl)piperidine-1-carboxylate **9** (100 mg, 0.30 mmol, 1.0 eq.), Et_3_N (84 µL, 0.60 mmol, 2.0 eq.) and bromomethylbenzene (39 µL, 0.33 mmol, 1.1 eq.) in CH_2_Cl_2_ according to the representative procedure **(E)** and stirring the reaction for 15 h at room temperature. The crude was purified by flash chromatography on silica gel (cyclohexane/EtOAc, gradient 100:0 to 80:20) to give **12** as a yellow oil (25 mg, 26% yield over two steps); ^1^H NMR (CDCl_3_-*d*, 400 MHz) *δ* 7.64 (dt, *^3^J* = 7.9 Hz, *^4^J* = *^5^J* = 1.1 Hz, 1H), 7.52 (ddd, *^3^J* = 8.4 Hz, *^3^J* = 7.0 Hz, *^4^J* = 1.2 Hz, 1H), 7.42 (dt, *^3^J* = 8.4 Hz, *^4^J* = *^5^J* = 0.9 Hz, 1H), 7.34–7.27 (m, 5H), 7.26 (m, 1H), 4.30 (d, *^3^J* = 6.5 Hz, 2H), 3.57 (s, 2H), 2.99 (m, 2H), 2.07 (m, 2H), 1.97 (m, 1H), 1.85 (m, 2H), 1.52 (m, 2H); ^13^C NMR (CDCl_3_-*d*, 100 MHz) *δ* 166.8, 164.0, 130.5, 129.5 (2C), 128.4 (2C), 127.3, 123.0, 121.0, 114.5, 110.3, 74.7, 63.4, 53.2 (2C), 35.6, 29.9, 28.7 (2C); LC-MS (ESI) t_R_ = 3.54 min; *m/z* [M+H]^+^ 323.54; IR (neat, cm^-1^) *ν* 2930, 2856, 1644, 1386, 1116, 1029. Spectroscopic data consistent with that reported in the literature.**^[^****^[[6]](#footnote-6)]^**

**3-[(1-isobutyl-4-piperidyl)methoxy]-1,2-benzoxazole (13).** The compound was prepared from *tert*-butyl 4-(1,2-benzoxazol-3-yloxymethyl)piperidine-1-carboxylate **9** (60 mg, 0.18 mmol, 1.0 eq.), Et_3_N (50 µL, 0.36 mmol, 2.0 eq.) and 1-iodo-2-methyl-propane (23 µL, 0.20 mmol, 1.1 eq.) in EtOH according to the representative procedure **(E)** and stirring the reaction for 48 h at reflux. The crude was purified by flash chromatography on silica gel (cyclohexane/EtOAc, gradient 100:0 to 80:20) to give **13** as a yellow oil (12 mg, 23% yield over two steps); ^1^H NMR (CDCl_3_-*d*, 400 MHz) *δ* 7.65 (dt, *^3^J* = 7.9 Hz, *^4^J* = *^5^J* = 1.0 Hz, 1H), 7.52 (ddd, *^3^J* = 8.4 Hz, *^3^J* = 7.0 Hz, *^4^J* = 1.2 Hz, 1H), 7.43 (dt, *^3^J* = 8.5 Hz, *^4^J* = *^5^J* = 1.0 Hz, 1H), 7.26 (ddd, *^3^J* = 7.8 Hz, *^3^J* = 7.1 Hz, *^4^J* = 0.8 Hz, 1H), 4.30 (d, ^3^*J* = 6.6 Hz, 2H), 2.93 (m, 2H), 2.10 (d, ^3^*J* = 7.2 Hz, 2H), 1.97–1.92 (m, 3H), 1.84–1.77 (m, 3H), 1.48 (m, 2H), 0.90 (d, ^3^*J* = 6.2 Hz, 6H); ^13^C NMR (CDCl_3_-*d*, 100 MHz) *δ* 166.7, 163.9, 130.4, 122.9, 120.9, 114.4, 110.2, 74.8, 67.3, 53.7 (2C), 35.7, 28.8 (2C), 25.6, 21.1 (2C); LC-MS (ESI) t_R_ = 3.30 min; *m/z* [M+H]^+^ 289.58; HRMS/ESI: *m/z* calcd. for C_17_H_25_N_2_O_2_ [M+H]^+^ 289.1911, found 289.1911; IR (neat, cm^-1^) *ν* 2956, 2928, 2871, 2782–2737, 1614, 1538, 1446, 1361, 1240, 1158, 1107, 986, 920, 742.

**3-[[1-(cyclohexylmethyl)-4-piperidyl]methoxy]-4-methoxy-1,2-benzoxazole (14).** The compound was prepared from *tert*-butyl 4-[(4-methoxy-1,2-benzoxazol-3-yl)oxymethyl]piperidine-1-carboxylate **10** (90 mg, 0.25 mmol, 1.0 eq.), bromomethylcyclohexane (43 µL, 0.31 mmol, 1.25 eq.) and K_2_CO_3_ (346 mg, 2.5 mmol, 10.0 eq.) in DMF according to the representative procedure **(E)** and stirring the reaction for 3 h at 110 °C. The crude was purified by flash chromatography on silica gel (cyclohexane/EtOAc, gradient 100:0 to 80:20) to give **11** as a pale brown solid (40 mg, 45% yield over two steps); mp 79 °C; ^1^H NMR (CDCl_3_-*d*, 400 MHz) *δ* 7.41 (t, ^3^*J* = 8.2 Hz, 1H), 7.00 (d, ^3^*J* = 8.4 Hz, 1H), 6.59 (d, ^3^*J* = 8.0 Hz, 1H), 4.27 (d, ^3^*J* = 6.9 Hz, 2H), 3.95 (s, 3H), 2.90 (m, 2H), 2.11 (m, 2H), 1.97 (m, 1H), 1.94–1.65 (m, 9H), 1.49 (m, 1H), 1.47–1.37 (m, 2H), 1.27–1.10 (m, 3H), 0.87 (m, 2H); ^13^C NMR (CDCl_3_-*d*, 100 MHz) *δ* 166.5, 166.1, 155.1, 132.0, 104.4, 103.3, 103.0, 75.1, 66.3, 56.1, 54.0 (2C), 35.7, 35.4, 32.2 (2C), 29.0 (2C), 27.0, 26.4 (2C); LC-MS (ESI) t_R_ = 3.67 min; *m/z* [M+H]^+^ 359.65; HRMS/ESI: *m/z* calcd. for C_21_H_31_N_2_O_3_ [M+H]^+^ 359.2329, found 359.2328; IR (neat, cm^-1^) *ν* 2944, 2927, 2852, 2800, 2732, 1623, 1535, 1366, 1287, 1096, 987, 781, 736.

**3-[(1-benzyl-4-piperidyl)methoxy]-4-methoxy-1,2-benzoxazole (15).** The compound was prepared from *tert*-butyl 4-[(4-methoxy-1,2-benzoxazol-3-yl)oxymethyl]piperidine-1-carboxylate **10** (100 mg, 0.28 mmol, 1.0 eq.), Et_3_N (78 µL, 0.56 mmol, 2.0 eq.) and bromomethylbenzene (37 µL, 0.31 mmol, 1.1 eq.) in CH_2_Cl_2_ according to the representative procedure **(E)** and stirring the reaction for 15 h at room temperature. The crude was purified by flash chromatography on silica gel (cyclohexane/EtOAc, gradient 100:0 to 80:20) to give **15** as a pale yellow solid (85 mg, 86% yield over two steps); mp 67 °C; ^1^H NMR (CDCl_3_-*d*, 400 MHz) *δ* 7.41 (t, ^3^*J* = 8.2 Hz, 1H), 7.33–7.30 (m, 4H), 7.27–7.23 (m, 1H), 6.99 (d, ^3^*J* = 8.4 Hz, 1H), 6.59 (d, ^3^*J* = 8.0 Hz, 1H), 4.28 (d, ^3^*J* = 6.8 Hz, 2H), 3.94 (s, 3H), 3.52 (s, 2H), 2.94 (m, 2H), 2.03 (m, 2H), 1.98 (m, 1H), 1.85 (m, 2H), 1.44 (m, 2H); ^13^C NMR (CDCl_3_-*d*, 100 MHz) *δ* 166.5, 166.1, 155.1, 138.6, 132.0, 129.4 (2C), 128.3 (2C), 127.1, 104.4, 103.3, 103.0, 75.0, 63.6, 56.1, 53.3 (2C), 35.6, 28.9 (2C); LC-MS (ESI) t_R_ = 3.50 min; *m/z* [M+H]^+^ 353.60; HRMS/ESI: *m/z* calcd. for C_21_H_25_N_2_O_3_ [M+H]^+^ 353.1860, found 353.1859; IR (neat, cm^-1^) *ν* 2939, 2914, 2847, 2796, 2755, 1615, 1532, 1361, 1285, 1098, 985, 930, 784, 740.

**3-[(1-isobutyl-4-piperidyl)methoxy]-4-methoxy-1,2-benzoxazole (16).** The compound was prepared from *tert*-butyl 4-[(4-methoxy-1,2-benzoxazol-3-yl)oxymethyl]piperidine-1-carboxylate **10** (90 mg, 0.25 mmol, 1.0 eq.), Et_3_N (70 µL, 0.50 mmol, 2.0 eq.) and 1-iodo-2-methyl-propane (32 µL, 0.28 mmol, 1.1 eq.) in EtOH according to the representative procedure **(E)** and stirring the reaction for 48 h at reflux. The crude was purified by flash chromatography on silica gel (cyclohexane/EtOAc, gradient 100:0 to 80:20) to give **16** as a yellow oil (29 mg, 36% yield over two steps); ^1^H NMR (CDCl_3_-*d*, 400 MHz) *δ* 7.41 (t, ^3^*J* = 8.2 Hz, 1H), 6.99 (d, ^3^*J* = 8.4 Hz, 1H), 6.59 (d, ^3^*J* = 8.0 Hz, 1H), 4.28 (d, ^3^*J* = 6.8 Hz, 2H), 3.95 (s, 3H), 2.90 (m, 2H), 2.07 (d, ^3^*J* = 7.3 Hz, 2H), 1.97 (m, 1H), 1.92 (m, 2H), 1.85–1.75 (m, 3H), 1.43 (m, 2H), 0.89 (d, ^3^*J* = 6.6 Hz, 6H); ^13^C NMR (CDCl_3_-*d*, 100 MHz) *δ* 166.5, 166.1, 155.1, 132.0, 104.4, 103.3, 103.0, 75.1, 67.5, 56.1, 53.9 (2C), 35.7, 29.0 (2C), 25.7, 21.2 (2C); LC-MS (ESI) t_R_ = 3.26 min; *m/z* [M+H]^+^ 319.60; HRMS/ESI: *m/z* calcd. for C_18_H_27_N_2_O_3_ [M+H]^+^ 319.2016, found 319.2013; IR (neat, cm^-1^) *ν* 2952, 2870, 2806, 2778, 1614, 1533, 1425, 1364, 1282, 1102, 983, 790, 742.

**(*E,Z*)-1-(2-bromophenyl)ethanone oxime (18a-b).** The compound was prepared from 1-(2-bromophenyl)ethenone **17** (2.08 mL, 15.4 mmol, 1.0 eq.) according to the representative procedure **(F)** and refluxing the reaction for 2 h. A mixture of (*Z*)-isomer **18a**/ (*E*)-isomer **18b** (1:2) was obtained as a white solid (2.98 g, 90% yield); HRMS/ESI: *m/z* calcd. for C_8_H_9_BrNO [M+H]^+^ 213.9862, found 213.9857; IR (neat, cm^-1^) *ν* 3232, 2920, 1426, 1311, 1026, 926, 756; **(*Z*)-isomer 18a** ^1^H NMR (CDCl_3_-*d*, 400 MHz) *δ* 8.22 (br s, 1H), 7.64 (dd, ^3^*J* = 8.0 Hz, ^4^*J* = 1.1 Hz, 1H), 7.39 (td, ^3^*J* = 7.5 Hz, ^4^*J* = 1.1 Hz, 1H), 7.25 (m, 1H), 7.17 (dd, ^3^*J* = 7.6 Hz, ^4^*J* = 1.6 Hz, 1H), 2.29 (s, 3H, CH_3_); ^13^C NMR (CDCl_3_-*d*, 100 MHz) *δ* 155.7, 137.3, 132.9, 130.0, 128.2, 127.6, 120.0, 21.2; **(*E*)-isomer 18b** ^1^H NMR (CDCl_3_-*d*, 400 MHz) *δ* 8.86 (br s, 1H), 7.61 (dd, ^3^*J* = 8.1 Hz, ^4^*J* = 1.1 Hz, 1H), 7.34 (td, ^3^*J* = 7.7 Hz, ^4^*J* = 1.1 Hz, 1H), 7.33 (m, 1H), 7.25 (m, 1H), 2.23 (s, 3H); ^13^C NMR (CDCl_3_-*d*, 100 MHz) *δ* 158.1, 138.8, 133.3, 130.3, 130.2, 127.5, 121.9, 16.1. Spectroscopic data consistent with that reported in the literature.**^[^^[[7]](#footnote-7)]^**

**(*E*,*Z*)-1-(2,6-dihydroxyphenyl)ethanone oxime (25a-b).** The compound was prepared from 1-(2,6-dihydroxyphenyl)ethenone **24** (5.0 g, 32.9 mmol, 1.0 eq.) according to the representative procedure **(F)** and refluxing the reaction for 3 h. The crude was purified by chromatography on silica gel column (cyclohexane/EtOAc, gradient 100:0 to 90:10) to isolate (*E*)-isomer **25b**, as a white solid (4.40 g, 80% yield) after concentration *in vacuo*, and then (cyclohexane/EtOAc, gradient 90:10 to 80:20) to isolate (*Z*)-isomer **25a**, as a white solid (1.10 g, 20% yield) after concentration *in vacuo*; ; **(*Z*)-isomer 25a** ^1^H NMR (MeOD-*d_4_*, 400 MHz) *δ* 6.99 (t, *^3^J* = 8.1 Hz, 1H), 6.36 (d, *^3^J* = 8.1 Hz, 2H), 2.13 (s, 3H); ^13^C NMR (MeOD-*d_4_*, 100 MHz) *δ* 155.9 (2C), 154.3, 130.9, 112.3, 108.2 (2C), 20.4; **(*E*)-isomer 25b** mp 183 °C; ^1^H NMR (MeOD-*d_4_*, 400 MHz) *δ* 6.95 (t, *^3^J* = 8.2 Hz, 1H), 6.33 (d, *^3^J* = 8.2 Hz, 2H), 2.28 (s, 3H); ^13^C NMR (MeOD-*d_4_*, 100 MHz) *δ* 158.2 (2C), 157.9, 130.9, 111.6, 108.1 (2C), 15.5; IR (neat, cm^-1^) *ν* 3306, 1621, 1465, 1366, 1284, 989, 896, 783, 585. Spectroscopic data consistent with that reported in the literature.**^[^****^[[8]](#footnote-8)]^**

**3-methyl-1,2-benzoxazole (19).** To a stirred and degassed solution of a mixture of (*Z*)-isomer **18a**/(*E*)-isomer **18b** (1:2) (2.88 g, 13.5 mmol, 1.0 eq.) in dry THF (30 mL) was added *t*-BuONa (2.59 g, 27 mmol, 2.0 eq.). The resulting mixture was flushed with nitrogen and stirred at room temperature for 30 min. DMEDA (436 µL, 4.05 mmol, 30 mol%) was added and the mixture was stirred for 5 min, before CuI (257 mg, 1.35 mmol, 10 mol%) was added. The resulting mixture was stirred at room temperature for 1 h and diluted with CH_2_Cl_2_. The organic layer was washed with 1N HCl, water and brine, then dried over MgSO_4_ and concentrated *in vacuo*. The crude was purified by chromatography on silica gel column (cyclohexane/EtOAc, gradient 100:0 to 95:5) to afford the compound **19** as a colorless oil (480 mg, 27% yield); ^1^H NMR (CDCl_3_-*d*, 400 MHz) *δ* 7.64 (dt, *^3^J* = 7.9 Hz, *^4^J* = *^5^J* = 1.0 Hz, 1H), 7.56–7.54 (m, 2H), 7.31 (ddd, *^3^J* = 7.9 Hz, *^3^J* = 4.8 Hz, *^4^J* = 3.1 Hz, 1H), 2.59 (s, 3H); ^13^C NMR (CDCl_3_-*d*, 100 MHz) *δ* 163.0, 155.1, 129.9, 123.3, 122.4, 121.3, 110.0, 10.3; IR (neat, cm^-1^) *ν* 2963, 2925, 2852, 1611, 1528, 1446, 1392, 1237, 1018, 897, 856, 751. Spectroscopic data consistent with that reported in the literature.**^[7]^**

***Tert*-butyl 4-[2-(1,2-benzoxazol-3-yl)ethyl]piperidine-1-carboxylate (20).** The compound was prepared from 3-methyl-1,2-benzoxazole **19** (480 mg, 3.6 mmol, 1.0 eq.) and 1.0 M LDA solution (3.6 mL, 3.6 mmol, 1.0 eq.) according to the representative procedure **(G)**. The crude was purified by flash chromatography on silica gel (cyclohexane/EtOAc, gradient 100:0 to 80:20) to give **20** as a pale yellow solid (257 mg, 22% isolated yield); mp 57 °C; ^1^H NMR (CDCl_3_-*d*, 400 MHz) *δ* 7.65 (dt, *^3^J* = 7.9 Hz, *^4^J* = *^5^J* = 1.0 Hz, 1H), 7.58–7.52 (m, 2H), 7.31 (ddd, *^3^J* = 7.9 Hz, *^3^J* = 5.5 Hz, *^4^J* = 2.4 Hz, 1H), 4.11 (m, 2H), 3.03 (t, ^3^*J* = 7.8 Hz, 2H), 2.69 (m, 2H), 1.85–1.75 (m, 4H), 1.53 (m, 1H), 1.47 (s, 9H), 1.18 (m, 2H); ^13^C NMR (CDCl_3_-*d*, 100 MHz) *δ* 163.0, 158.4, 154.9, 129.8, 123.2, 121.6, 121.2, 110.0, 79.3, 43.7 (2C), 35.5, 34.1, 31.9 (2C), 28.5 (3C), 22.6; IR (neat, cm^-1^) *ν* 2975, 2935, 2851, 1684, 1612, 1417, 1232, 1162, 754. Spectroscopic data consistent with that reported in the literature.**^[7]^**

**3-[2-[1-(cyclohexylmethyl)-4-piperidyl]ethyl]-1,2-benzoxazole (21).** The compound was prepared from *tert*-butyl 4-[2-(1,2-benzoxazol-3-yl)ethyl]piperidine-1-carboxylate **20** (80 mg, 0.24 mmol, 1.0 eq.), bromomethylcyclohexane (50 µL, 0.36 mmol, 1.5 eq.) and K_2_CO_3_ (332 mg, 2.40 mmol, 10.0 eq.) in DMF according to the representative procedure **(E)** and stirring the reaction for 3 h at 110 °C. The crude was purified by flash chromatography on silica gel (cyclohexane/EtOAc, gradient 100:0 to 80:20) to give **21** as a pale yellow solid (39 mg, 50% yield over two steps); mp 62 °C; ^1^H NMR (CDCl_3_-*d*, 399.8 MHz) *δ* 7.65 (dt, *^3^J* = 7.9 Hz, *^4^J* = *^5^J* = 1.0 Hz, 1H), 7.58–7.49 (m, 2H), 7.30 (ddd, *^3^J* = 7.9 Hz, *^3^J* = 6.0 Hz, *^4^J* = 2.0 Hz, 1H), 3.00 (t, ^3^*J* = 7.9 Hz, 2H), 2.86 (m, 2H), 2.08 (d, ^3^*J* = 7.0 Hz, 2H), 1.86–1.64 (m, 11H), 1.47 (m, 1H), 1.35–1.10 (m, 6H), 0.86 (m, 2H); ^13^C NMR (CDCl_3_-*d*, 100 MHz) *δ* 163.1, 158.8, 129.8, 123.2, 121.8, 121.4, 110.1, 66.4, 54.5 (2C), 35.7, 35.4, 34.5, 32.3 (4C), 27.0, 26.4 (2C), 22.9; LC-MS (ESI) t_R_ = 3.65 min; *m/z* [M+H]^+^ 327.55; HRMS/ESI: *m/z* calcd. for C_21_H_31_N_2_O [M+H]^+^ 327.2431, found 327.2430; IR (neat, cm^-1^) *ν* 2919, 2849, 2796, 1612, 1446, 1234, 1131, 870, 750.

**3-[2-(1-benzyl-4-piperidyl)ethyl]-1,2-benzoxazole (22).** The compound was prepared from *tert*-butyl 4-[2-(1,2-benzoxazol-3-yl)ethyl]piperidine-1-carboxylate **20** (80 mg, 0.24 mmol, 1.0 eq.), Et_3_N (72 µL, 0.56 mmol, 2.0 eq.) and bromomethylbenzene (31 µL, 0.26 mmol, 1.1 eq.) in CH_2_Cl_2_ according to the representative procedure **(E)** and stirring the reaction for 15 h at room temperature. The crude was purified by flash chromatography on silica gel (cyclohexane/EtOAc, gradient 100:0 to 80:20) to give **22** as a yellow oil (29 mg, 38% yield over two steps); ^1^H NMR (CDCl_3_-*d*, 400 MHz) *δ* 7.64 (dt, *^3^J* = 7.9 Hz, *^4^J* = *^5^J* = 1.0 Hz, 1H), 7.55–7.53 (m, 2H), 7.32–7.22 (m, 6H), 3.50 (s, 2H), 3.00 (t, ^3^*J* = 7.8 Hz, 2H), 2.90 (m, 2H), 1.95 (m, 2H), 1.83–1.74 (m, 4H), 1.39–1.32 (m, 3H); ^13^C NMR (CDCl_3_-*d*, 100 MHz) *δ* 163.1, 158.8, 138.5, 129.8, 129.4 (2C), 128.3 (2C), 127.1, 123.2, 121.8, 121.4, 110.1, 63.6, 53.8 (2C), 35.4, 34.4, 32.2 (2C), 22.9; LC-MS (ESI) t_R_ = 3.51 min; *m/z* [M+H]^+^ 321.44; IR (neat, cm^-1^) *ν* 2923, 2851, 2803, 2760, 1608, 1439, 1237, 1130, 749, 697. Spectroscopic data consistent with that reported in the literature.**^[7]^**

**3-[2-(1-isobutyl-4-piperidyl)ethyl]-1,2-benzoxazole (23).** The compound was prepared from *tert*-butyl 4-[2-(1,2-benzoxazol-3-yl)ethyl]piperidine-1-carboxylate **20** (75 mg, 0.23 mmol, 1.0 eq.), Et_3_N (64 µL, 0.46 mmol, 2.0 eq.) and 1-iodo-2-methyl-propane (32 µL, 0.28 mmol, 1.2 eq.) in EtOH according to the representative procedure **(E)** and stirring the reaction for 48 h at reflux. The crude was purified by flash chromatography on silica gel (cyclohexane/EtOAc, gradient 100:0 to 70:30) to give **23** as a yellow oil (10 mg, 15% yield over two steps); ^1^H NMR (CDCl_3_-*d*, 400 MHz) *δ* 7.65 (dt, *^3^J* = 7.9 Hz, *^4^J* = *^5^J* = 1.2 Hz, 1H), 7.57–7.51 (m, 2H), 7.30 (ddd, *^3^J* = 7.9 Hz, *^3^J* = 5.9 Hz, *^4^J* = 1.9 Hz, 1H), 3.01 (t, ^3^*J* = 7.8 Hz, 2H), 2.88 (m, 2H), 2.06 (d, ^3^*J* = 7.3 Hz, 2H), 1.89–1.74 (m, 7H), 1.40–1.28 (m, 3H), 0.89 (d, *^3^J* = 6.6 Hz, 6H); ^13^C NMR (CDCl_3_-*d*, 100 MHz) *δ* 163.1, 158.8, 129.8, 123.2, 121.8, 121.4, 110.1, 67.5, 54.4 (2C), 35.6, 34.5, 32.2 (2C), 25.7, 22.9, 21.2 (2C); LC-MS (ESI) t_R_ = 3.46 min; *m/z* [M+H]^+^ 287.61; HRMS/ESI: *m/z* calcd. for C_18_H_27_N_2_O [M+H]^+^ 287.2118, found 287.2117; IR (neat, cm^-1^) *ν* 2951, 2923, 2870, 2795, 2776, 1610, 1437, 1382, 1239, 1096, 750.

**[(*E*)-1-(2,6-dihydroxyphenyl)ethylideneamino] acetate (26).** Acetic anhydride (11 mL) was added to (*E*)-1-(2,6-dihydroxyphenyl)ethanone oxime **25b** (3.33 g, 19.9 mmol, 1.0 eq.)**.** The resulting mixture was stirred at room temperature for 1 h, then concentrated *in vacuo* (use of cyclohexane for the co-evaporation of Ac_2_O). Water was added and the solid obtained was filtered, washed with water and concentrated under reduced pressure to afford the compound **26** as a white solid (3.46 g, 83% yield); mp 157 °C; ^1^H NMR (MeOD-*d_4_*, 400 MHz) *δ* 7.04 (t, *^3^J* = 8.2 Hz, 1H), 6.37 (d, *^3^J* = 8.2 Hz, 2H), 2.36 (s, 3H), 2.22 (s, 3H); ^13^C NMR (MeOD-*d_4_*, 100 MHz) *δ* 170.2, 165.6, 158.0 (2C), 132.2, 111.1, 108.1 (2C), 19.3, 17.7; IR (neat, cm^-1^) *ν* 3243, 1721, 1619, 1460, 1230, 986, 899, 792, 741. Spectroscopic data consistent with that reported in the literature.**^[9]^**

**2-methyl-1,3-benzoxazol-4-ol (27) & 3-methyl-1,2-benzoxazol-4-ol (28).** [(*E*)-1-(2,6-dihydroxy phenyl)ethylideneamino] acetate **26** (550 mg, 2.2 mmol, 1.0 eq.) was dissolved in pyridine (20 mL) and the resulting mixture was stirred at reflux for 1 h. After cooling to room temperature, the reaction mixture was poured over 1 N HCl solution. The aqueous layer was extracted several times with EtOAc and the organic layer was washed with additional 1 N HCl and brine, dried over MgSO_4_ and then concentrated *in vacuo.* The crude was purified by chromatography on silica gel column (cyclohexane/EtOAc, gradient 100:0 to 98:2) and concentrated under reduced pressure to afford a mixture **27**/**28** 30:70 as a white solid (1.02 g, 71% yield); HRMS/ESI: *m/z* calcd. for C_8_H_8_NO_2_ [M+H]^+^ 150.0550, found 150.0548. **Compound 27** ^1^H NMR (MeOD-*d_4_*, 400 MHz) *δ* 7.13 (t, *^3^J* = 8.1 Hz, 1H), 6.99 (dd, *^3^J* = 8.2 Hz, *^4^J* = 0.9 Hz, 1H), 6.71 (dd, *^3^J* = 8.1 Hz, *^4^J* = 0.9 Hz, 1H), 2.59 (s, 3H); ^13^C NMR (MeOD-*d_4_*, 100 MHz) *δ* 164.0, 154.0, 150.1, 130.7, 126.4, 111.0, 102.4, 14.0. **Compound 28** ^1^H NMR (MeOD-*d_4_*, 400 MHz) *δ* 7.34 (dd, *^3^J* = 8.3 Hz, *^3^J* = 7.9 Hz, 1H), 6.95 (dd, *^3^J* = 8.3 Hz, *^4^J* = 0.6 Hz, 1H), 6.58 (dd, *^3^J* = 7.8 Hz, *^4^J* = 0.6 Hz, 1H), 2.62 (s, 3H); ^13^C NMR (MeOD-*d_4_*, 100 MHz) *δ* 166.4, 156.1, 155.3, 132.9, 112.7, 108.4, 101.2, 11.8.

**4-methoxy-2-methyl-1,3-benzoxazole (29a) & 4-methoxy-3-methyl-1,2-benzoxazole (30a).** Compounds were prepared from a mixture **27**/**28** 3:7 (400 mg, 2.68 mmol, 1.0 eq.) according to the representative procedure **(H)** and stirring the reaction for 3 h at 80 °C. The crude was purified by flash chromatography on silica gel (cyclohexane/EtOAc, gradient 100:0 to 98:2) to isolate **30a** as a white solid (204 mg, 47% yield) after concentration *in vacuo*, and then (cyclohexane/EtOAc, gradient 98:2 to 95:5) to isolate **29a** as a pale yellow solid (102 mg, 23% yield) after concentration *in vacuo*. **Compound 29a** mp 79 °C; ^1^H NMR (CDCl_3_-*d*, 400 MHz) *δ* 7.41 (t, *^3^J* = 8.2 Hz, 1H), 7.09 (d, *^3^J* = 8.4 Hz, 1H), 6.59 (d, *^3^J* = 8.0 Hz, 1H), 3.93 (s, 3H), 2.64 (s, 3H); ^13^C NMR (CDCl_3_-*d*, 100 MHz) *δ* 165.0, 155.6, 154.9, 131.4, 112.5, 102.9, 102.6, 55.7, 12.1; HRMS/ESI: *m/z* calcd. for C_9_H_10_NO_2_ [M+H]^+^ 164.0706, found 164.0704; IR (neat, cm^-1^) *ν* 2952, 2923, 2845, 1608, 1501, 1394, 1281, 1100, 877, 781, 737. **Compound 30a** mp 63 °C; ^1^H NMR (CDCl_3_-*d*, 400 MHz) *δ* 7.20 (t, ^3^*J* = 8.2 Hz, 1H), 7.08 (d, ^3^*J* = 8.2 Hz, 1H), 6.75 (d, ^3^*J* = 8.1 Hz, 1H), 4.00 (s, 3H), 2.61 (s, 3H); ^13^C NMR (CDCl_3_-*d*, 100 MHz) *δ* 162.6, 152.5, 151.2, 131.1, 125.1, 105.9, 103.2, 56.3, 14.5; MS *m/z* [M+H]^+^ 164.37; HRMS/ESI: *m/z* calcd. for C_9_H_10_NO_2_ [M+H]^+^ 164.0706, found 164.0702; IR (neat, cm^-1^) *ν* 2967, 2943, 2845, 1624, 1504, 1264, 1090, 779, 738.

**4-ethoxy-2-methyl-1,3-benzoxazole (29b) & 4-ethoxy-3-methyl-1,2-benzoxazole (30b).** Compounds were prepared from a mixture **27**/**28** 3:7 (235 mg, 1.58 mmol, 1.0 eq.) according to the representative procedure **(H)** and stirring the reaction for 15 h at room temperature. The crude was purified by chromatography on silica gel column (cyclohexane/EtOAc, gradient 100:0 to 98:2) to isolate **30b** as a white solid (98 mg, 35% yield) after concentration *in vacuo*, and then (cyclohexane/EtOAc, gradient 98:2 to 95:5) to isolate **29b** as a colorless oil (63 mg, 23% yield) after concentration *in vacuo*. **Compound 29b** ^1^H NMR (CDCl_3_-*d*, 400 MHz) *δ* 7.19 (t, *^3^J* = 8.2 Hz, 1H), 7.07 (dd, *^3^J* = 8.4 Hz, *^4^J* = 0.8 Hz, 1H), 6.74 (dd, *^3^J* = 8.1 Hz, *^4^J* = 0.8 Hz, 1H), 4.26 (q, *^3^J* = 7.0 Hz, 2H), 2.62 (s, 3H), 1.51 (t, *^3^J* = 7.0 Hz, 3H); ^13^C NMR (CDCl_3_-*d*, 100 MHz) *δ* 162.5, 152.6, 150.5, 131.1, 125.1, 106.8, 103.0, 64.6, 15.0, 14.5; HRMS/ESI: *m/z* calcd. for C_10_H_12_NO_2_ [M+H]^+^ 178.0863, found 178.0864; IR (neat, cm^-1^) *ν* 2980, 2922, 2883, 1615, 1498, 1254, 1084, 781, 742. **Compound 30b** mp 86 °C; ^1^H NMR (CDCl_3_-*d*, 400 MHz) *δ* 7.39 (t, *^3^J* = 8.2 Hz, 1H), 7.07 (d, *^3^J* = 8.4 Hz, 1H), 6.57 (d, *^3^J* = 8.0 Hz, 1H), 4.15 (q, *^3^J* = 7.0 Hz, 2H), 2.65 (s, 3H), 1.49 (t, *^3^J* = 7.0 Hz, 3H); ^13^C NMR (CDCl_3_-*d*, 100 MHz) *δ* 165.0, 155.0 (2C), 131.4, 112.6, 103.5, 102.4, 64.1, 14.8, 12.0; HRMS/ESI: *m/z* calcd. for C_10_H_12_NO_2_ [M+H]^+^ 178.0863, found 178.0862; IR (neat, cm^-1^) *ν* 2978, 2930, 2900, 1606, 1501, 1390, 1285, 1091, 783, 745.

**4-isobutoxy-2-methyl-1,3-benzoxazole (29c) & 4-isobutoxy-3-methyl-1,2-benzoxazole (30c).** Compounds were prepared from a mixture **27**/**28** 3:7 (250 mg, 1.68 mmol, 1.0 eq.) according to the representative procedure **(H)** and stirring the reaction for 15 h at room temperature. The crude was purified by chromatography on silica gel column (cyclohexane/EtOAc, gradient 100:0 to 98:2) to isolate **30c** as a white solid (75 mg, 22% yield) after concentration *in vacuo*, and then (cyclohexane/EtOAc, gradient 98:2 to 95:5) to isolate **29c** as a yellow oil (35 mg, 10% yield) after concentration *in vacuo*. **Compound 29c** ^1^H NMR (CDCl_3_-*d*, 400 MHz) *δ* 7.18 (t, *^3^J* = 8.2 Hz, 1H), 7.06 (dd, *^3^J* = 8.2 Hz, *^4^J* = 0.8 Hz, 1H), 6.74 (dd, *^3^J* = 8.2 Hz, *^4^J* = 0.7 Hz, 1H), 3.95 (d, *^3^J* = 6.7 Hz, 2H), 2.62 (s, 3H), 2.23 (m, 1H), 1.07 (d, *^3^J* = 6.7 Hz, 6H); ^13^C NMR (CDCl_3_-*d*, 100 MHz) *δ* 162.4, 152.6, 150.8, 131.1, 125.0, 107.1, 102.9, 75.6, 28.3, 19.5 (2C), 14.6; HRMS/ESI: *m/z* calcd. for C_12_H_16_NO_2_ [M+H]^+^ 206.1176, found 206.1179; IR (neat, cm^-1^) *ν* 2959, 2935, 2873, 1621, 1501, 1257, 1088, 734. **Compound 30c** mp <50 °C; ^1^H NMR (CDCl_3_-*d*, 400 MHz) *δ* 7.38 (t, *^3^J* = 8.2 Hz, 1H), 7.06 (d, *^3^J* = 8.4 Hz, 1H), 6.55 (d, *^3^J* = 8.0 Hz, 1H), 3.85 (d, *^3^J* = 6.3 Hz, 2H), 2.66 (s, 3H), 2.17 (m, 1H), 1.08 (d, *^3^J* = 6.7 Hz, 6H); ^13^C NMR (CDCl_3_-*d*, 100 MHz) *δ* 165.0, 155.1, 154.9, 131.4, 112.6, 103.4, 102.2, 74.6, 28.4, 19.4 (2C), 12.2; HRMS/ESI: *m/z* calcd. for C_12_H_16_NO_2_ [M+H]^+^ 206.1176, found 206.1174; IR (neat, cm^-1^) *ν* 2965, 2933, 2871, 1611, 1501, 1287, 1098, 778, 736.

**4-(cyclopentoxy)-2-methyl-1,3-benzoxazole (29d) & 4-(cyclopentoxy)-3-methyl-1,2-benzoxazole (30d).** Compounds were prepared from a mixture **27**/**28** 3:7 (290 mg, 1.94 mmol, 1.0 eq.) according to the representative procedure **(H)** and stirring the reaction for 48 h at room temperature. The crude was purified by chromatography on silica gel column (cyclohexane/EtOAc, gradient 100:0 to 98:2) to isolate **30d** as a white solid (229 mg, 54% yield) after concentration *in vacuo*, and then (cyclohexane/EtOAc, gradient 98:2 to 95:5) to isolate **29d** as a yellow oil (105 mg, 25% yield) after concentration *in vacuo*. **Compound 29d** ^1^H NMR (CDCl_3_-*d*, 400 MHz) *δ* 7.18 (t, *^3^J* = 8.2 Hz, 1H), 7.05 (dd, *^3^J* = 8.2 Hz, *^4^J* = 0.8 Hz, 1H), 6.74 (d, *^3^J* = 8.1 Hz, 1H), 5.04 (qt, *^3^J* = 4.5 Hz, 1H), 2.62 (s, 3H), 2.01–1.96 (m, 4H), 1.92–1.81 (m, 2H), 1.67–1.57 (m, 2H); ^13^C NMR (CDCl_3_-*d*, 100 MHz) *δ* 162.4, 152.7, 149.8, 131.5, 125.0, 108.3, 102.6, 80.5, 33.1 (2C), 24.4 (2C), 14.6; HRMS/ESI: *m/z* calcd. for C_13_H_16_NO_2_ [M+H]^+^ 218.1176, found 218.1174; IR (neat, cm^-1^) *ν* 3042, 2961, 2873, 1620, 1498, 1256, 1072, 781, 736. **Compound 30d** mp <50 °C; ^1^H NMR (CDCl_3_-*d*, 400 MHz) *δ* 7.38 (t, *^3^J* = 8.2 Hz, 1H), 7.04 (d, *^3^J* = 8.4 Hz, *^4^J* = 0.5 Hz, 1H), 6.57 (d, *^3^J* = 8.0 Hz, 1H), 4.91 (qt, *^3^J* = 4.0 Hz, 1H), 2.62 (s, 3H), 1.97–1.93 (m, 4H), 1.88–1.78 (m, 2H), 1.74–1.64 (m, 2H); ^13^C NMR (CDCl_3_-*d*, 100 MHz) *δ* 165.1, 155.0, 154.0, 131.3, 113.0, 104.7, 101.9, 80.0, 33.0 (2C), 24.2 (2C), 12.1; HRMS/ESI: *m/z* calcd. for C_13_H_16_NO_2_ [M+H]^+^ 218.1176, found 218.1174; IR (neat, cm^-1^) *ν* 2961, 2871, 1610, 1496, 1279, 1085, 774, 729.

**4-benzyloxy-2-methyl-1,3-benzoxazole (29e) & 4-benzyloxy-3-methyl-1,2-benzoxazole (30e).** Compounds were prepared from a mixture **27**/**28** 3:7 (1.0 g, 6.7 mmol, 1.0 eq.) according to the representative procedure **(H)** and stirring the reaction for 15 h at room temperature. The crude was purified by chromatography on silica gel column (cyclohexane/EtOAc, gradient 100:0 to 98:2) to isolate **30e** as a white solid (1.09 g, 68% yield) after concentration *in vacuo*, and then (cyclohexane/EtOAc, gradient 98:2 to 95:5) to isolate **29e** as a white solid (475 mg, 30% yield) after concentration *in vacuo*. **Compound 29e** mp 61 °C; ^1^H NMR (CDCl_3_-*d*, 400 MHz) *δ* 7.48 (m, 2H), 7.38-7.25 (m, 3H), 7.15 (t, *^3^J* = 8.1 Hz, 1H), 7.08 (d, *^3^J* = 8.1 Hz, 1H), 6.78 (d, *^3^J* = 8.0 Hz, 1H), 5.35 (s, 2H), 2.62 (s, 3H); ^13^C NMR (CDCl_3_-*d*, 100 MHz) *δ* 162.5, 152.6, 150.1, 136.9, 131.2, 128.6 (2C), 128.0, 127.6 (2C), 124.9, 107.9, 103.3, 71.1, 14.5; HRMS/ESI: *m/z* calcd. for C_15_H_14_NO_2_ [M+H]^+^ 240.1019, found 240.1019; IR (neat, cm^-1^) *ν* 3089, 2930, 2868, 1620, 1495, 1261, 1072, 747, 702. **Compound 30e** mp 88 °C; ^1^H NMR (CDCl_3_-*d*, 400 MHz) *δ* 7.48–7.35 (m, 6H), 7.12 (d, *^3^J* = 8.4 Hz, 1H), 6.68 (d, *^3^J* = 7.9 Hz, 1H), 5.20 (s, 2H), 2.66 (s, 3H); ^13^C NMR (CDCl_3_-*d*, 100 MHz) *δ* 165.1, 154.9, 154.5, 136.3, 131.4, 128.8 (2C), 128.3, 127.3 (2C), 112.8, 104.1, 102.9, 70.4, 12.2; HRMS/ESI: *m/z* calcd. for C_15_H_14_NO_2_ [M+H]^+^ 240.1019, found 240.1018; IR (neat, cm^-1^) *ν* 3066, 2926, 2874, 1618, 1501, 1287, 1094, 738, 716.

***Tert*-butyl 4-[2-(4-methoxy-1,2-benzoxazol-3-yl)ethyl]piperidine-1-carboxylate (31a).** The compound was prepared from 4-methoxy-3-methyl-1,2-benzoxazole **30a** (190 mg, 1.2 mmol, 1.0 eq.) and 1.0 M LDA solution (1.2 mL, 1.2 mmol, 1.0 eq.) according to the representative procedure **(G)**. The crude was purified by flash chromatography on silica gel (cyclohexane/EtOAc, gradient 100:0 to 90:10) to give **31a** as a yellow oil (193 mg, 45% yield); ^1^H NMR (CDCl_3_-*d*, 400 MHz) *δ* 7.43 (t, *^3^J* = 8.2 Hz, 1H), 7.11 (d, *^3^J* = 8.4 Hz, 1H), 6.61 (d, *^3^J* = 8.0 Hz, 1H), 4.10 (m, 2H), 3.95 (s, 3H), 3.05 (t, *^3^J* = 7.8 Hz, 2H), 2.70 (m, 2H), 1.79–1.74 (m, 4H), 1.50 (m, 1H), 1.46 (s, 9H), 1.17 (m, 2H); ^13^C NMR (CDCl_3_-*d*, 100 MHz) *δ* 165.0, 158.4, 155.1, 154.9, 131.3, 111.9, 102.9, 102.6, 79.3, 55.6, 43.8 (2C), 35.6, 34.6, 31.9 (2C), 28.5 (3C), 23.8; HRMS/ESI: *m/z* calcd. for C_20_H_29_N_2_O_4_ [M+H]^+^ 361.2122, found 361.2122; IR (neat, cm^-1^) *ν* 2967, 2929, 2850, 1691, 1611, 1424, 1365, 1279, 1162, 1102.

***Tert*-butyl 4-[2-(4-ethoxy-1,2-benzoxazol-3-yl)ethyl]piperidine-1-carboxylate (31b).** The compound was prepared from 4-ethoxy-3-methyl-1,2-benzoxazole **30b** (90 mg, 0.51 mmol, 1.0 eq.) and 1.0 M LDA solution (510 µL, 0.51 mmol, 1.0 eq.) according to the representative procedure **(G)**. The crude was purified by flash chromatography on silica gel (cyclohexane/EtOAc, gradient 100:0 to 90:10) to give **31b** as a yellow oil (52 mg, 27% yield); ^1^H NMR (CDCl_3_-*d*, 400 MHz) *δ* 7.40 (t, *^3^J* = 8.1 Hz, 1H), 7.08 (d, *^3^J* = 8.3 Hz, 1H), 6.58 (d, *^3^J* = 7.9 Hz, 1H), 4.17 (q, *^3^J* = 7.0 Hz, 2H), 4.09 (m, 2H), 3.05 (t, *^3^J* = 7.8 Hz, 2H), 2.69 (m, 2H), 1.80–1.71 (m, 4H), 1.51 (m, 1H), 1.49 (t, *^3^J* = 7.0 Hz, 3H),1.45 (s, 9H), 1.16 (m, 2H); ^13^C NMR (CDCl_3_-*d*, 100 MHz) *δ* 165.0, 158.5, 154.9, 154.4, 131.3, 111.8, 103.5, 102.3, 79.3, 64.0, 44.0 (2C), 35.8, 34.8, 32.0 (2C), 28.5 (3C), 23.8, 14.8; HRMS/ESI: *m/z* calcd. for C_21_H_31_N_2_O_4_ [M+H]^+^ 375.2278, found 375.2278; IR (neat, cm^-1^) *ν* 2977, 2931, 2857, 1692, 1611, 1424, 1365, 1281, 1162, 1092.

***Tert*-butyl 4-[2-(4-isobutoxy-1,2-benzoxazol-3-yl)ethyl]piperidine-1-carboxylate (31c).** The compound was prepared from 4-isobutoxy-3-methyl-1,2-benzoxazole **30c** (70 mg, 0.34 mmol, 1.0 eq.) and 1.0 M LDA solution (340 µL, 0.34 mmol, 1.0 eq.) according to the representative procedure **(G)**. The crude was purified by flash chromatography on silica gel (cyclohexane/EtOAc, gradient 100:0 to 90:10) to give **31c** as a yellow oil (29 mg, 21% yield); ^1^H NMR (CDCl_3_-*d*, 400 MHz) *δ* 7.40 (t, *^3^J* = 8.0 Hz, 1H), 7.09 (d, *^3^J* = 8.2 Hz, 1H), 6.58 (d, *^3^J* = 7.8 Hz, 1H), 4.09 (m, 2H), 3.87 (d, *^3^J* = 6.4 Hz, 2H), 3.08 (t, *^3^J* = 7.8 Hz, 2H), 2.68 (m, 2H), 2.17 (m, 1H), 1.82–1.72 (m, 4H), 1.50 (m, 1H), 1.45 (s, 9H), 1.15 (m, 2H), 1.09 (d, *^3^J* = 6.8 Hz, 6H); ^13^C NMR (CDCl_3_-*d*, 100 MHz) *δ* 165.1, 158.5, 155.0, 154.8, 131.4, 112.0, 103.6, 102.4, 79.4, 74.8, 44.0 (2C), 35.8, 34.7, 32.1 (2C), 28.6 (3C), 28.4, 24.1, 19.5 (2C); HRMS/ESI: *m/z* calcd. for C_23_H_35_N_2_O_4_ [M+H]^+^ 403.2591, found 403.2590; IR (neat, cm^-1^) *ν* 2967, 2929, 2863, 1692, 1610, 1424, 1281, 1162, 1094.

***Tert*-butyl 4-[2-[4-(cyclopentoxy)-1,2-benzoxazol-3-yl]ethyl]piperidine-1-carboxylate (31d).** The compound was prepared from 4-(cyclopentoxy)-3-methyl-1,2-benzoxazole **30d** (215 mg, 1.0 mmol, 1.0 eq.) and 1.0 M LDA solution (1.0 mL, 1.0 mmol, 1.0 eq.) according to the representative procedure **(G)**. The crude was purified by flash chromatography on silica gel (cyclohexane/EtOAc, gradient 100:0 to 90:10) to give **31d** as a yellow oil (103 mg, 25% yield); ^1^H NMR (CDCl_3_-*d*, 400 MHz) *δ* 7.38 (t, *^3^J* = 8.1 Hz, 1H), 7.05 (d, *^3^J* = 8.3 Hz, 1H), 6.57 (d, *^3^J* = 8.0 Hz, 1H), 4.92 (m, 1H), 4.10 (m, 2H), 3.02 (t, *^3^J* = 7.8 Hz, 2H), 2.68 (m, 2H), 2.00–1.89 (m, 4H), 1.85–1.66 (m, 8H), 1.49 (m, 1H), 1.45 (s, 9H), 1.14 (m, 2H); ^13^C NMR (CDCl_3_-*d*, 100 MHz) *δ* 165.3, 158.6, 155.1, 153.7, 131.4, 112.4, 104.8, 102.0, 80.1, 79.4, 44.1 (2C), 36.0, 35.0, 33.1 (2C), 32.2 (2C), 28.7 (3C), 24.3 (2C), 24.1; HRMS/ESI: *m/z* calcd. for C_24_H_35_N_2_O_4_ [M+H]^+^ 415.2591, found 415.2590; IR (neat, cm^-1^) *ν* 2967, 2931, 2860, 1692, 1609, 1424, 1365, 1278, 1162, 1082.

***Tert*-butyl 4-[2-(4-benzyloxy-1,2-benzoxazol-3-yl)ethyl]piperidine-1-carboxylate (31e).** The compound was prepared from 4-benzyloxy-3-methyl-1,2-benzoxazole **30e** (1.07 g, 4.5 mmol, 1.0 eq.) and 1.0 M LDA solution (4.5 mL, 4.5 mmol, 1.0 eq.) according to the representative procedure **(G)**. The crude was purified by flash chromatography on silica gel (cyclohexane/EtOAc, gradient 100:0 to 90:10) to give **31e** as a colorless oil (930 mg, 47% yield); ^1^H NMR (CDCl_3_-*d*, 400 MHz) *δ* 7.47–7.39 (m, 6H), 7.14 (d, *^3^J* = 8.3 Hz, 1H), 6.71 (d, *^3^J* = 7.9 Hz, 1H), 5.17 (s, 2H), 4.00 (m, 2H), 2.98 (t, *^3^J* = 8.0 Hz, 2H), 2.56 (m, 2H), 1.70–1.62 (m, 2H), 1.52 (m, 2H), 1.47 (s, 9H), 1.29 (m, 1H), 1.15 (m, 2H); ^13^C NMR (CDCl_3_-*d*, 100 MHz) *δ* 165.2, 158.6, 154.9, 154.3, 136.0, 131.5, 128.9 (2C), 128.7, 128.2 (2C), 112.1, 104.0, 103.0, 79.3, 70.7, 44.0 (2C), 35.9, 35.0, 31.8 (2C), 28.6 (3C), 24.1; HRMS/ESI: *m/z* calcd. for C_26_H_33_N_2_O_4_ [M+H]^+^ 437.2435, found 437.2435; IR (neat, cm^-1^) *ν* 2926, 2855, 1688, 1615, 1423, 1280, 1163, 1091.

**3-[2-[1-(cyclohexylmethyl)-4-piperidyl]ethyl]-4-methoxy-1,2-benzoxazole (32a).** The compound was prepared from *tert*-butyl 4-[2-(4-methoxy-1,2-benzoxazol-3-yl)ethyl]piperidine-1-carboxylate **31a** (180 mg, 0.50 mmol, 1.0 eq.), bromomethylcyclohexane (91 µL, 0.65 mmol, 1.3 eq.) and K_2_CO_3_ (898 mg, 6.50 mmol, 10.0 eq.) in DMF according to the representative procedure **(E)** and stirring the reaction for 3 h at 110 °C. The crude was purified by flash chromatography on silica gel (cyclohexane/EtOAc, gradient 100:0 to 50:50) to give **32a** as a yellow oil (119 mg, 67% yield over two steps); ^1^H NMR (CDCl_3_-*d*, 400 MHz) *δ* 7.42 (dd, *^3^J* = *^3^J* = 8.0 Hz, 1H), 7.10 (dd, *^3^J* = 8.2 Hz, *^4^J* = 0.5 Hz, 1H), 6.61 (dd, *^3^J* = 7.8 Hz, *^3^J* = 0.5 Hz, 1H), 3.94 (s, 3H), 3.03 (t, *^3^J* = 7.9 Hz, 2H), 2.87 (m, 2H), 2.09 (d, *^3^J* = 7.0 Hz, 2H), 1.84 (m, 2H), 1.77–1.63 (m, 9H), 1.49 (m, 1H), 1.35–1.10 (m, 6H), 0.87 (m, 2H); ^13^C NMR (CDCl_3_-*d*, 100 MHz) *δ* 165.0, 158.8, 155.3, 131.3, 112.1, 102.9, 102.6, 66.5, 55.7, 54.7 (2C), 35.7, 35.4, 35.0, 32.3 (4C), 26.9, 26.4 (2C), 24.1; LC-MS (ESI) t_R_ = 3.85 min; *m/z* [M+H]^+^ 357.60; HRMS/ESI: *m/z* calcd. for C_22_H_33_N_2_O_2_ [M+H]^+^ 357.2537, found 357.2535; IR (neat, cm^-1^) *ν* 2921, 2849, 2802, 2766, 1611, 1499, 1280, 1103, 785, 729.

**3-[2-[1-(cyclohexylmethyl)-4-piperidyl]ethyl]-4-ethoxy-1,2-benzoxazole (32b).** The compound was prepared from *tert*-butyl 4-[2-(4-ethoxy-1,2-benzoxazol-3-yl)ethyl]piperidine-1-carboxylate **31b** (50 mg, 0.13 mmol, 1.0 eq.), bromomethylcyclohexane (24 µL, 0.17 mmol, 1.3 eq.) and K_2_CO_3_ (180 mg, 1.30 mmol, 10.0 eq.) in DMF according to the representative procedure **(E)** and stirring the reaction for 2 h at 110 °C. The crude was purified by flash chromatography on silica gel (cyclohexane/EtOAc, gradient 100:0 to 50:50) to give **32b** as a yellow oil (11 mg, 23% yield over two steps); ^1^H NMR (CDCl_3_-*d*, 400 MHz) *δ* 7.40 (dd, *^3^J* = *^3^J* = 8.2 Hz, 1H), 7.08 (dd, *^3^J* = 8.4 Hz, *^4^J* = 0.5 Hz, 1H), 6.58 (d, *^3^J* = 8.0 Hz, 1H), 4.17 (q, *^3^J* = 7.0 Hz, 2H), 3.04 (t, *^3^J* = 7.9 Hz, 2H), 2.86 (m, 2H), 2.09 (d, *^3^J* = 7.0 Hz, 2H), 1.84 (m, 2H), 1.78–1.63 (m, 9H), 1.50 (t, *^3^J* = 7.0 Hz, 3H), 1.48 (m, 1H), 1.35–1.10 (m, 6H), 0.86 (m, 2H); ^13^C NMR (CDCl_3_-*d*, 100 MHz) *δ* 165.1, 158.9, 154.6, 131.3, 112.1, 103.5, 102.4, 66.5, 64.1, 54.7 (2C), 36.0, 35.4, 35.1, 32.3 (4C), 27.0, 26.4 (2C), 24.2, 14.9; LC-MS (ESI) t_R_ = 4.09 min; *m/z* [M+H]^+^ 371.61; HRMS/ESI: *m/z* calcd. for C_23_H_35_N_2_O_2_ [M+H]^+^ 371.2693, found 371.2693; IR (neat, cm^-1^) *ν* 2921, 2851, 2799, 2764, 1611, 1282, 1111, 1092, 788, 733.

**3-[2-[1-(cyclohexylmethyl)-4-piperidyl]ethyl]-4-isobutoxy-1,2-benzoxazole (32c).** The compound was prepared from *tert*-butyl 4-[2-(4-isobutoxy-1,2-benzoxazol-3-yl)ethyl]piperidine-1-carboxylate **31c** (32 mg, 0.08 mmol, 1.0 eq.), bromomethylcyclohexane (15 µL, 0.10 mmol, 1.3 eq.) and K_2_CO_3_ (138 mg, 1.00 mmol, 10.0 eq.) in DMF according to the representative procedure **(E)** and stirring the reaction for 2 h at 110 °C. The crude was purified by flash chromatography on silica gel (cyclohexane/EtOAc, gradient 100:0 to 50:50) to give **32c** as a yellow oil (10 mg, 31% yield over two steps); ^1^H NMR (CDCl_3_-*d*, 400 MHz) *δ* 7.39 (t, *^3^J* = 8.1 Hz, 1H), 7.08 (d, *^3^J* = 8.3 Hz, 1H), 6.58 (d, *^3^J* = 7.9 Hz, 1H), 3.87 (d, *^3^J* = 6.4 Hz, 2H), 3.07 (t, *^3^J* = 7.8 Hz, 2H), 2.86 (m, 2H), 2.18 (m, 1H), 2.09 (d, *^3^J* = 6.9 Hz, 2H), 1.87–1.64 (m, 11H), 1.48 (m, 1H), 1.33–1.12 (m, 6H), 1.09 (d, *^3^J* = 6.7 Hz, 6H), 0.86 (m, 2H); ^13^C NMR (CDCl_3_-*d*, 100 MHz) *δ* 165.1, 158.8, 154.8, 131.3, 112.0, 103.5, 102.4, 74.8, 66.4, 54.6 (2C), 35.9, 35.4, 34.9, 32.3 (4C), 28.4, 27.0, 26.4 (2C), 24.3, 19.5 (2C); LC-MS (ESI) t_R_ = 4.44 min; *m/z* [M+H]^+^ 399.66; HRMS/ESI: *m/z* calcd. for C_25_H_39_N_2_O_2_ [M+H]^+^ 399.3006, found 399.3005; IR (neat, cm^-1^) *ν* 2925, 2854, 2798, 1613, 1467, 1450, 1385, 1282, 1100, 746.

**3-[2-[1-(cyclohexylmethyl)-4-piperidyl]ethyl]-4-(cyclopentoxy)-1,2-benzoxazole (32d).** The compound was prepared from *tert*-butyl 4-[2-[4-(cyclopentoxy)-1,2-benzoxazol-3-yl]ethyl]piperidine-1-carboxylate **31d** (100 mg, 0.24 mmol, 1.0 eq.), bromomethylcyclohexane (44 µL, 0.31 mmol, 1.3 eq.) and K_2_CO_3_ (428 mg, 3.1 mmol, 10.0 eq.) in DMF according to the representative procedure **(E)** and stirring the reaction for 2 h at 110 °C. The crude was purified by flash chromatography on silica gel (cyclohexane/EtOAc, gradient 100:0 to 50:50) to give **32d** as a yellow oil (30 mg, 30% yield over two steps); ^1^H NMR (CDCl_3_-*d*, 400 MHz) *δ* 7.39 (t, *^3^J* = 8.2 Hz, 1H), 7.05 (dd, *^3^J* = 8.3 Hz, *^3^J* = 0.4 Hz, 1H), 6.57 (d, *^3^J* = 8.0 Hz, 1H), 4.93 (m, 1H), 3.01 (t, *^3^J* = 7.9 Hz, 2H), 2.86 (m, 2H), 2.09 (d, *^3^J* = 7.0 Hz, 2H), 1.99–1.91 (m, 4H), 1.86–1.64 (m, 15H), 1.48 (m, 1H), 1.33–1.10 (m, 6H), 0.86 (m, 2H); ^13^C NMR (CDCl_3_-*d*, 100 MHz) *δ* 165.2, 158.9, 153.7, 131.2, 112.4, 104.6, 101.9, 80.0, 66.4, 54.7 (2C), 36.1, 35.4, 35.1, 33.0 (2C), 32.3 (4C), 27.0, 26.4 (2C), 24.3, 24.2 (2C); LC-MS (ESI) t_R_ = 4.37 min; *m/z* [M+H]^+^ 411.67; HRMS/ESI: *m/z* calcd. for C_26_H_39_N_2_O_2_ [M+H]^+^ 411.3006, found 411.3006; IR (neat, cm^-1^) *ν* 2921, 2851, 2796, 2760, 1609, 1355, 1278, 1083, 788.

**4-benzyloxy-3-[2-[1-(cyclohexylmethyl)-4-piperidyl]ethyl]-1,2-benzoxazole (32e).** The compound was prepared from *tert*-butyl 4-[2-(4-benzyloxy-1,2-benzoxazol-3-yl)ethyl]piperidine-1-carboxylate **31e** (580 mg, 1.33 mmol, 1.0 eq.), bromomethylcyclohexane (279 µL, 2.00 mmol, 1.5 eq.) and K_2_CO_3_ (1.84 mg, 13.3 mmol, 10.0 eq.) in DMF according to the representative procedure **(E)** and stirring the reaction for 6 h at 110 °C. The crude was purified by flash chromatography on silica gel (cyclohexane/EtOAc, gradient 100:0 to 50:50) to give **32e** as a pale yellow solid (452 mg, 79% yield over two steps); mp 96 °C; ^1^H NMR (CDCl_3_-*d*, 400 MHz) *δ* 7.47–7.33 (m, 6H), 7.12 (d, *^3^J* = 8.3 Hz, 1H), 6.69 (d, *^3^J* = 7.9 Hz, 1H), 5.18 (s, 2H), 3.00 (t, *^3^J* = 8.3 Hz, 2H), 2.76 (m, 2H), 2.05 (d, *^3^J* = 7.1 Hz, 2H), 1.76–1.65 (m, 9H), 1.54 (m, 2H), 1.46 (m, 1H), 1.28–1.08 (m, 6H), 0.85 (m, 2H); ^13^C NMR (CDCl_3_-*d*, 100 MHz) *δ* 165.1, 158.9, 154.3, 136.1, 131.3, 128.8 (2C), 128.5, 127.9 (2C), 112.2, 104.0, 102.9, 70.6, 66.4, 54.6 (2C), 35.9, 35.4, 35.3, 32.2 (2C), 32.1 (2C), 27.0, 26.4 (2C), 24.3; LC-MS (ESI) t_R_ = 4.23 min; *m/z* [M+H]^+^ 433.67; HRMS/ESI: *m/z* calcd. for C_28_H_37_N_2_O_2_ [M+H]^+^ 433.2850, found 433.2849; IR (neat, cm^-1^) *ν* 2919, 2849, 2793, 2760, 1612, 1451, 1375, 1283, 1089, 755, 712.

**3-[2-[1-(cyclohexylmethyl)-4-piperidyl]ethyl]-1,2-benzoxazol-4-ol (33).** To a stirred solution of 4-benzyloxy-3-[2-[1-(cyclohexylmethyl)-4-piperidyl]ethyl]-1,2-benzoxazole **32e** (380 mg, 0.88 mmol, 1.0 eq.) was added a hydrobromic acid solution (33 wt.% in acetic acid, 45 mL) at 0°C. The resulting mixture was stirred at 50 °C for 3 h, and was poured slowly into a mixture water/ice. The residue was then extracted with EtOAc. The organic layer was washed several times with brine, dried over MgSO_4_ and concentrated *in vacuo*. The crude was taken up in CH_2_Cl_2_ and the solid obtained was filtered and concentrated under reduced pressure to afford the compound **33** as a white solid (256 mg, 85% yield); mp 231 °C; ^1^H NMR (MeOD-*d_4_*, 400 MHz) *δ* 7.37 (dd, *^3^J* = 8.3 Hz, *^3^J* = 7.9 Hz, 1H), 6.98 (dd, *^3^J* = 8.3 Hz, *^4^J* = 0.5 Hz, 1H), 6.61 (dd, *^3^J* = 7.9 Hz, *^3^J* = 0.5 Hz, 1H), 3.57 (m, 2H), 3.12 (t, *^3^J* = 7.5 Hz, 2H), 2.94 (m, 4H), 2.07 (m, 2H), 1.91–1.67 (m, 8H), 1.66–1.53 (m, 3H), 1.42–1.17 (m, 3H), 1.05 (m, 2H); ^13^C NMR (MeOD-*d_4_*, 100 MHz) *δ* 166.5, 159.4, 154.9, 133.0, 112.1, 108.5, 101.4, 64.6, 54.6 (2C), 34.8, 34.1 (2C), 31.8 (2C), 30.3 (2C), 26.9, 26.5 (2C), 24.3; LC-MS (ESI) t_R_ = 3.64 min; *m/z* [M+H]^+^ 343.57; HRMS/ESI: *m/z* calcd. for C_21_H_31_N_2_O_2_ [M+H]^+^ 343.2380, found 343.2379; IR (neat, cm^-1^) *ν* 3116, 2932, 2852, 2730, 2663, 1613, 1433, 1353, 1290, 1067, 795.

**Crystallographic data of compounds 7, 25b, 27, 30a and 30e**


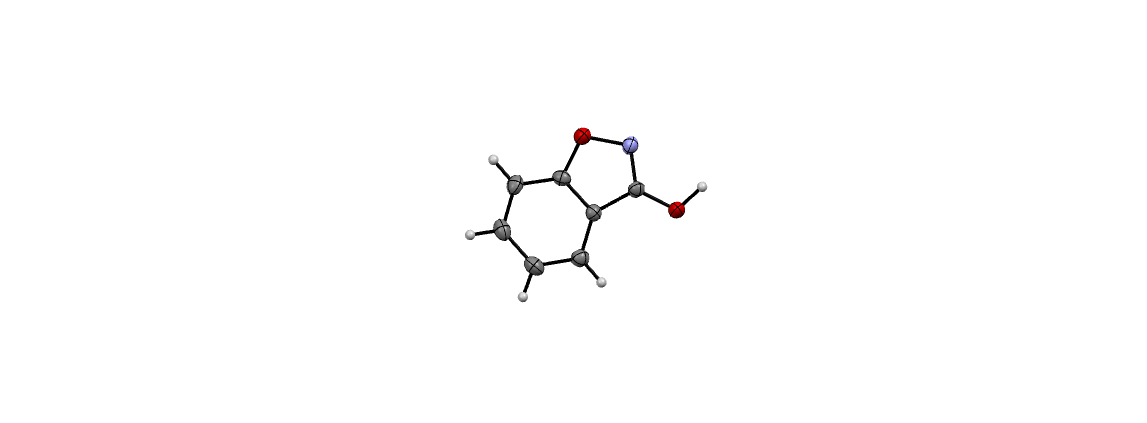


**1,2-benzoxazol-3-ol (7).** RX (ORTEP). Crystal system monoclinic, space group P 2_1_/n, *a* = 5.6606(5) Å, *b* = 6.5947(6) Å, *c* = 16.2359(17) Å, α = 90°, β = 95.166(6)°, γ = 90°, V = 603.62(10) Å^3^, Z = 4, calculated density = 1.487 g/cm^3^, μ = 0.11 mm^–1^, R_int_ = 0.044, R[*F*2>2σ(*F*2)] = 0.050, wR(*F*2) = 0.116.


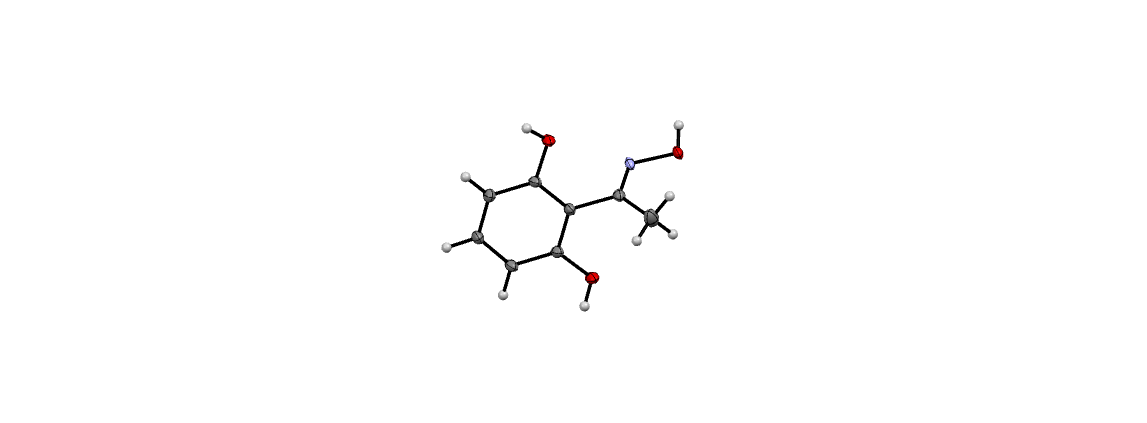


**(*E*)-1-(2,6-dihydroxyphenyl)ethanone oxime (25b).** RX (ORTEP). Crystal system orthorhombic, space group P 2_1_ 2_1_ 2_1_, *a* = 6.8218(3) Å, *b* = 7.5936(4) Å, *c* = 14.6877(7) Å, α = 90°, β = 90°, γ = 90°, V = 760.85(6) Å^3^, Z = 4, calculated density = 1.459 g/cm^3^, μ = 0.11 mm^–1^, R_int_ = 0.050, R[*F*2>2σ(*F*2)] = 0.035, wR(*F*2) = 0.096.


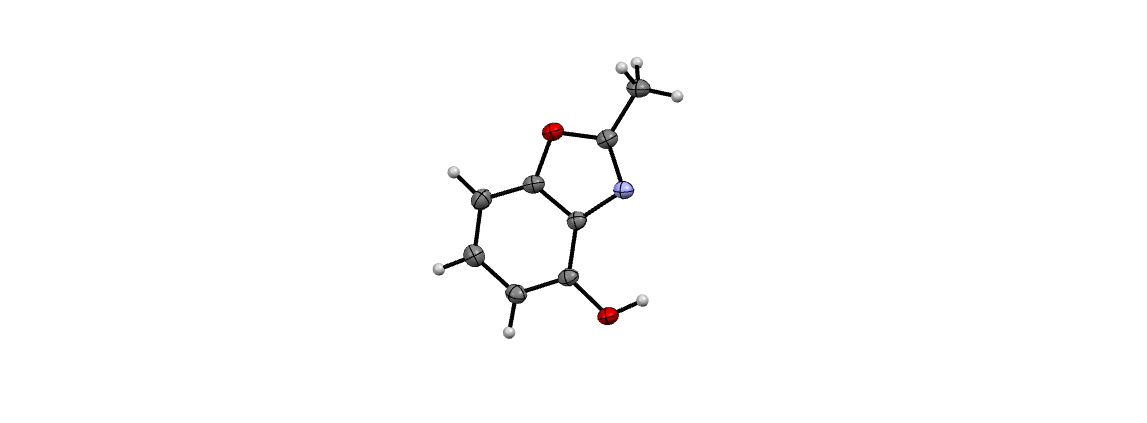


**2-methyl-1,3-benzoxazol-4-ol (27).** RX (ORTEP). Crystal system triclinic, space group P-1, *a* = 5.3112(2) Å, *b* = 7.8910(3) Å, *c* = 17.4960(6) Å, α = 92.782(2)°, β = 94.301(2)°, γ = 108.321(2)°, V = 692.12(4) Å^3^, Z = 4, calculated density = 1.431 g/cm^3^, μ = 0.11 mm^–1^, R_int_ = 0.033, R[*F*2>2σ(*F*2)] = 0.041, wR(*F*2) = 0.107.

**4-methoxy-3-methyl-1,2-benzoxazole (30a).** RX (ORTEP). Crystal system orthorhombic, space group P c a 2_1_, *a* = 14.4563(4) Å, *b* = 6.0225(2) Å, *c* = 8.9909(3) Å, α = 90°, β = 90°, γ = 90°, V = 782.78(4) Å^3^, Z = 4, calculated density = 1.385 g/cm^3^, μ = 0.10 mm^–1^, R_int_ = 0.054, R[*F*2>2σ(*F*2)] = 0.044, wR(*F*2) = 0.088.


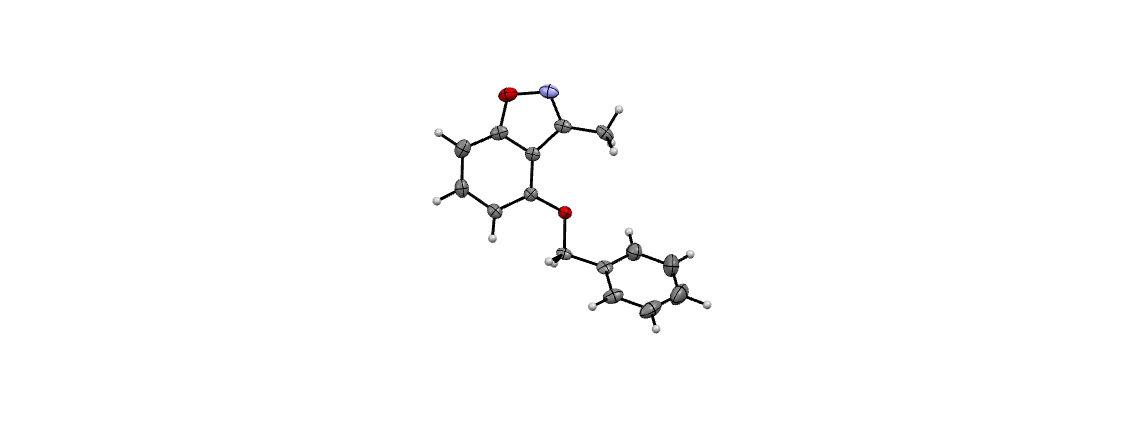


**4-benzyloxy-3-methyl-1,2-benzoxazole (30e).** RX (ORTEP). Crystal system orthorhombic, space group P b c a, *a* = 7.4003(3) Å, *b* = 14.5972(8) Å, *c* = 22.6995(11) Å, α = 90°, β = 90°, γ = 90°, V = 2452.1(2) Å^3^, Z = 12, calculated density = 1.432 g/cm^3^, μ = 0.10 mm^–1^, R_int_ = 0.067, R[*F*2>2σ(*F*2)] = 0.127, wR(*F*2) = 0.209.

**5-HT4 pharmacological profile determination**

**Compound 16**

*Pharmacological profile: not agonist*

|  | **EC50** | **Log(EC50)** | **Top AMPc** |
| --- | --- | --- | --- |
| **HP5** | ns | ns | 5.63 % |
| **HP6** | ns | ns | 0.00 % |
| **HP7** | ns | ns | 6.91 % |
| **Moy.** | **ns** | **ns** | **4 %** |
| **SEM** |  |  | **2 %** |

ns: not significant

*Pharmacological profile: antagonist*

|  | **IC50** | **Log(IC50)** | **Bott. AMPc** | **Basal** |
| --- | --- | --- | --- | --- |
| **HP8** | 5.07E-09 | -8.3 | 39.69 % | 28% |
| **HP10** | 2.01E-09 | -8.7 | 29.62 % | 29% |
| **HP11** | 9.78E-09 | -8.0 | 24.98 % | 29% |
| **Moy.** | **5.62E-09** | **-8.3** | **31 %** |  |
| **SEM** | **2.26E-09** | **0.2** | **4 %** |  |

**Compound 32a**

*Pharmacological profile: not agonist*

|  | **EC50** | **Log(EC50)** | **Top AMPc** |
| --- | --- | --- | --- |
| **HP5** | 1.36E-05 | -4.9 | 29.36 % |
| **HP6** | 2.83E-10 | -9.5 | 12.83 % |
| **HP7** | 2.03E-05 | -4.7 | 24.95 % |
| **Moy.** | **1.13E-05** | **-4.9** | **22 %** |
| **SEM** | **5.97E-06** | **1.6** | **5 %** |

*Pharmacological profile: antagonist*

|  | **IC50** | **Log(IC50)** | **Bott. AMPc** | **Basal** |
| --- | --- | --- | --- | --- |
| **HP8** | 1.29E-07 | -6.9 | 38.80 % | 25% |
| **HP10** | 6.95E-08 | -7.2 | 27.69 % | 22% |
| **HP11** | 9.36E-08 | -7.0 | 32.50 % | 35% |
| **Moy.** | **9.72E-08** | **-7.0** | **33 %** |  |
| **SEM** | **1.72E-08** | **0.1** | **3 %** |  |

**Compound 33**

*Pharmacological profile: not agonist*

|  | **EC50** | **Log(EC50)** | **Top AMPc** |
| --- | --- | --- | --- |
| **HP5** | ns | ns | -15.21 % |
| **HP6** | ns | ns | 4.96 % |
| **HP7** | ns | ns | 0.00 % |
| **Moy.** | **ns** | **ns** | **-3 %** |
| **SEM** |  |  | **6 %** |

ns: not significant

*Pharmacological profile: antagonist*

|  | **IC50** | **Log(IC50)** | **Bott. AMPc** | **Basal** |
| --- | --- | --- | --- | --- |
| **HP8** | 8.82E-08 | -7.1 | 50.44 % | 29% |
| **HP10** | 2.05E-06 | -5.7 | 46.43 % | 26% |
| **HP11** | 5.07E-07 | -6.3 | 34.27 % | 32% |
| **Moy.** | **8.83E-07** | **-6.1** | **44 %** |  |
| **SEM** | **5.98E-07** | **0.4** | **5 %** |  |

**References**

**[1]** Sheldrick, G. M. Phase annealing in SHELX-​90: direct methods for larger structures. *Acta Crys* **A46**, 467–473, <https://doi.org/10.1107/S0108767390000277> (1990).

**[2]** Sheldrick, G. M. A short history of SHELX. *Acta Cryst* **A64**, 112–122, <https://doi.org/10.1107/S0108767307043930> (2008).

**[3]** Huang, S.-T. *et al.* Synthesis and Anticancer Evaluation of Bis(benzimidazoles), Bis(benzoxazoles), and Benzothiazoles. *Bioorg Med Chem* **14**, 6106–6119, <https://doi.org/10.1016/j.bmc.2006.05.007> (2006).

**[4]** Zhao, J. & Larock, R. C. Synthesis of Xanthones, Thioxanthones, and Acridones by the Coupling of Arynes and Substituted Benzoates. *J Org Chem* **72**, 583–588, <https://doi.org/10.1021/jo0620718> (2007).

**[5]** Lienard, B. M. R. *et al.* Inhibitors of the FEZ-1 Metallo-Beta-Lactamase. *Bioorg Med Chem Lett* **17**, 964–968, <https://doi.org/10.1016/j.bmcl.2006.11.053> (2007).

**[6]** Villalobos, A. *et al.* Novel Benzisoxazole Derivatives as Potent and Selective Inhibitors of Acetylcholinesterase. *J Med Chem* **37**, 2721–2734, <https://doi.org/10.1021/jm00043a012> (1994).

**[7]** Udd, S. *et al.* Copper-catalyzed cyclization of *Z*-oximes into 3-methyl-1,2-benzisoxazoles. *Tetrahedron Lett* **51**, 1030–1033, <https://doi.org/10.1016/j.tetlet.2009.12.070> (2010).

**[8]** Alvaro, G. *et al.* Hydantoin derivatives as Kv3 inhibitors. WO 2012/076877 A1, (2012).

1. **^[]^** Sheldrick, G. M. *Acta Cryst.* **1990**, *A46*(6), 467–473. [↑](#footnote-ref-1)
2. **^[]^** Sheldrick, G. M. *Acta Cryst.* **2008**, *A64*(1), 112–122. [↑](#footnote-ref-2)
3. **^[]^** Huang, S.-T.; Hsei, I.-J.; Chen, C. *Bioorg. Med. Chem.* **2006**, *14*, 6106–6119. [↑](#footnote-ref-3)
4. **^[]^** Zhao, J.; Larock, R. C. *J. Org. Chem.* **2007**, *72*, 583–588. [↑](#footnote-ref-4)
5. **^[]^** Lienard, B. M. R.; Horsfall, L. E.; Galleni, M.; Frere, J.-M.; Schofield, C. J. *Bioorg. Med. Chem. Lett.* **2007**, *17*, 964–968. [↑](#footnote-ref-5)
6. **^[]^** Villalobos, A.; Blake, J. F.; Biggers, C. K.; Butler, T. W.; Chapin, D. S.; Chen, Y. L.; Ives, J. L.; Jones, S. B.; Liston, D. R.; Nagel, A. A.; Nason, D. M.; Nielsen, J. A.; Shalaby, I. A.; White, W. F. *J. Med. Chem.* **1994**, *37*, 2721–2734. [↑](#footnote-ref-6)
7. **^[]^** Udd, S.; Jokela, R.; Franzén, R.; Tois, J. *Tetrahedron Lett.* **2010**, *51*, 1030–1033. [↑](#footnote-ref-7)
8. **^[]^** Alvaro, G.; Dambruoso, P.; Marasco, A.; Tommasi, S.; Decor, A.; Large, C. Preparation of hydantoin derivatives as Kv3 channel inhibitors. PCT Int. Appl., WO 2012/076877 A1 (GB2011/052414), **2012**. [↑](#footnote-ref-8)
